# A Dispersant‐Driven Carbon‐Binder Domains Homogeneity in High‐Energy‐Density Electrodes for Lithium‐Ion Batteries

**DOI:** 10.1002/advs.77293

**Published:** 2026-08-18

**Authors:** Da‐Sol Kwon, Min Young Seo, Jinho Ahn, Yea‐Ji Jeong, Woohyeon Shin, Wonseok Ko, Min Jeong Kim, Jiny Lee, Won‐Yong Jeon, Min‐Jae Choi, Kwang In Kim, Jin Ju Eom, Sang Yeop Oh, Honghee Lee, Joona Bang, Jongsoon Kim, YongJoo Kim, Jung‐Keun Yoo

**Affiliations:** ^1^ Energy Storage Research Center Korea Institute of Science and Technology (KIST) Seongbuk‐gu Seoul Republic of Korea; ^2^ Department of Chemical and Biological Engineering Korea University Seongbuk‐gu Seoul Republic of Korea; ^3^ Department of Materials Science and Engineering Korea University Seongbuk‐gu Seoul Republic of Korea; ^4^ Department of Energy Science Sungkyunkwan University Suwon Gyeonggi‐do Republic of Korea; ^5^ Department of Chemical and Biochemical Engineering Dongguk University Jung‐gu Seoul Republic of Korea; ^6^ Department of Energy Engineering Hanyang University Seongdong‐gu Seoul Republic of Korea; ^7^ Department of Advanced Materials Science and Engineering Sungkyunkwan University Suwon Gyeonggi‐do Republic of Korea; ^8^ Battery Materials R&D Center Hansol Chemical, Iksan‐si Jeonbuk‐do Republic of Korea; ^9^ Multiphysics Digital Twin Lab TRINITY Engineering Co., Ltd. Busan Republic of Korea; ^10^ SKKU Institute of Energy Science and Technology (SIEST) Sungkyunkwan University Suwon Gyeonggi‐do Republic of Korea

**Keywords:** carbon nanotube, carbon‐binder domain, dispersant, high energy density, lithium‐ion batteries

## Abstract

Achieving high energy density in lithium‐ion batteries (LIBs) requires electrodes with a high fraction of active materials. An extreme reduction of inactive components such as conductive additives and binders can limit electron transport and undermine electrode integrity, thereby deteriorating the electrochemical performance. To address these limitations, the formation of a well‐structured carbon‐binder domain (CBD) through effective dispersion of binder and conductive additives is crucial. Herein, we tune component compatibility in polyvinylidene fluoride (PVDF)–carbon nanotubes (CNTs) systems using an aromatic‐functionalized polyacrylate (AFPA), which enhances π–π interactions with CNTs and improve PVDF affinity. This approach promotes uniform binder distribution and enhances transport pathways, affecting the crystalline phase and reinforcing mechanical cohesion within the CBD. AFPA stabilizes PVDF in the *α*‐phase, whereas conventional hydrogenated nitrile butadiene rubber (HNBR) exhibits poor compatibility, leading to phase separation and *β*‐phase formation. This structural optimization facilitates interconnected electron/ion pathways and well‐connected pores under reduced inactive content and high mass loading. As a result, the AFPA‐based electrode delivers superior electrochemical performance even at a CNT content as low as 0.425 wt%. These findings establish CBD microstructural control as a key design parameter for achieving high‐energy‐density electrodes with minimized inactive content, providing a pathway toward next‐generation LIBs.

## Graphical abstract

A polar dispersant, aromatic‐functionalized polyacrylate (AFPA), enables the formation of a uniform CBD network by enhancing miscibility with PVDF and suppressing phase separation. This leads to *α*‐phase crystallization of PVDF and aligned CNT networks within the CBD, resulting in well‐connected structures that improve cohesion, electron/ion transport, and overall electrode performance in high‐energy cathode.

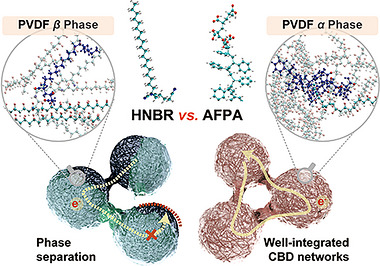

## 1 Introduction

The growing demand for longer‐range electric vehicles and portable electronics continues to drive the pursuit of higher energy density in lithium‐ion batteries [1, 2]. High‐energy‐density electrodes often employ higher active‐material content, inherently reducing the relative contents of binder and conductive additives [3, 4]. These reduced inactive‐component contents may shift electronic percolation toward the threshold and exacerbate spatial nonuniformities in the electrode microstructure, increasing overpotentials at high charge/discharge rates [5, 6, 7]. Such sensitivity highlights the importance of controlling microstructural uniformity in high‐energy‐density electrodes [6, 8].

As a key microstructural feature, the carbon–binder domain (CBD), composed of binder and conductive additives, governs electronic percolation, ionic transport, and the mechanical integrity of the electrode [9, 10, 11]. Structural inhomogeneity in the CBD is strongly influenced by dispersion quality during slurry mixing [12]. Specifically, when carbon nanotubes (CNTs) are used as conductive additives, their strong tendency to agglomerate can produce pronounced local carbon‐concentration gradients, thereby intensifying CBD inhomogeneity [13]. Uniform CNT dispersion in electrode slurries often relies on dispersant‐assisted dispersion systems [14].

Even when CNT dispersion is stabilized by dispersants, CBD uniformity can still be governed by the compatibility among CBD components including the binder, CNT, and the CNT dispersant. In industrial cathode manufacturing, hydrogenated nitrile butadiene rubber (HNBR) is widely used as a dispersant owing to its mechanical robustness and thermal stability [15, 16]. However, HNBR exhibits limited miscibility with polyvinylidene fluoride (PVDF) in N‐methyl‐2‐pyrrolidone (NMP)‐based slurries, which can manifest as phase separation under quiescent and low‐shear conditions during slurry preparation [17, 18, 19, 20]. Phase separation can disrupt CBD morphology and locally perturb PVDF crystallization, with binder redistribution during mixing and drying promoting an increased *β*‐phase fraction and, in turn, compromising CBD uniformity [21, 22].

A uniform CBD in PVDF/NMP slurries requires both dispersion stability and compatibility with the component to promote homogeneous mixing and suppress phase separation [15]. In this work, we employ an aromatic‐functionalized polyacrylate (AFPA) as a dispersant that enhances compatibility across CBD components. AFPA features a polar polyacrylate backbone that improves miscibility with both PVDF and NMP, and aromatic side groups that promote π–π interactions with CNTs. The dual functionality stabilizes dispersion and is associated with a shift in PVDF crystallization toward the *α*‐phase. The resulting uniform CBD microstructure can supports mechanical integrity and electrochemical stability.

As a result, AFPA‐based electrodes exhibit a highly interconnected CBD network with continuous electronic and ionic pathways, leading to significantly reduced internal resistance and enhanced electrochemical performance with as little as 0.21 wt% of AFPA. At a mass loading of 25 mg cm^−^^2^, AFPA‐based electrodes exhibit a 2 C capacity that is 1.7 times higher than that of HNBR‐based electrodes (160.3 vs. 94.0 mAh g^−^^1^). The higher capacity is consistent with reduced voltage polarization associated with improved CBD uniformity, which becomes increasingly important in thick, high‐loading electrodes under reduced conductive‐additive content [23]. Furthermore, the experimental trends are qualitatively consistent with digital twin prediction based on the characterized CBD microstructure. These findings establish CBD uniformity as a key design parameter governing microstructure–performance relationships in high‐energy‐density electrodes [24].

## 2 Results and Discussion

### 2.1 Dispersant‐Directed Structuring of CBD for Enhanced Network Connectivity

**FIGURE 1:**
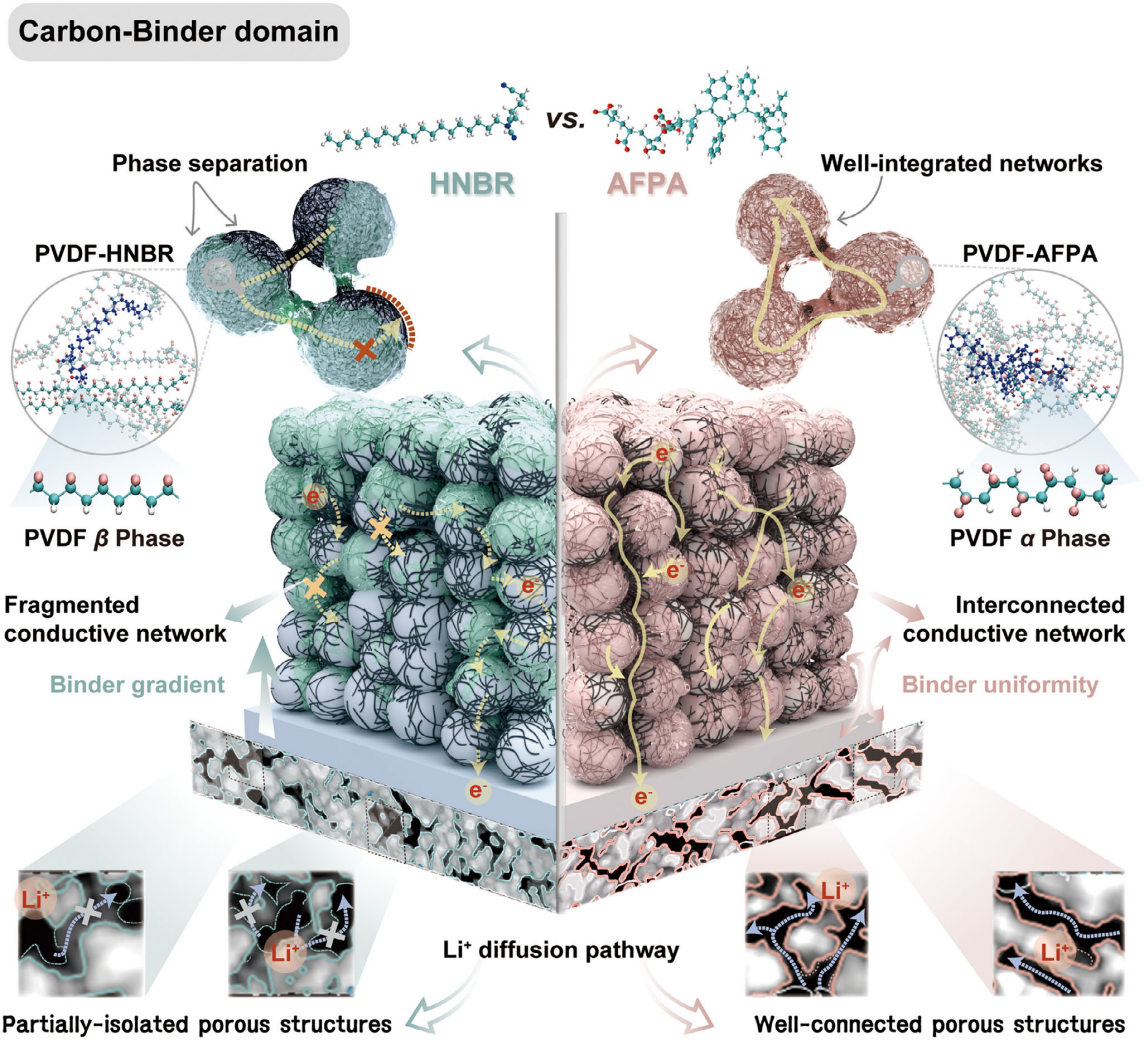
Schematic illustration depicting how dispersant‐induced CBD morphology governs binder crystalline phase and distribution, thereby affecting ion and electron transport networks.

Figure 1 schematically illustrates how the choice of dispersant influences CBD structure and binder crystallization behavior. Even in small amounts, the dispersant markedly alters electrode morphology and governs key CBD characteristics, including electron /ion transport pathways and binder distribution. These effects originate from differences in compatibility with PVDF and NMP, as evidenced by phase separation in PVDF–HNBR but not in PVDF–AFPA (Figure S1). Phase separation in HNBR‐based electrodes promotes local PVDF chain alignment, which in turn shifts PVDF crystallization toward the *β*‐phase [21, 22]. The resulting nonuniform binder distribution disrupts CBD continuity, leading to a fragmented microstructure and less efficient transport pathways. In contrast, AFPA suppresses phase separation by stabilizing both CNT and PVDF domain in NMP. Its dual functionality integrates a polar backbone that enhances miscibility with PVDF and the polar solvent NMP, and aromatic groups that promote π–π interactions with CNTs. This synergistic interaction improves compatibility in AFPA‐based electrodes, thereby promoting a more homogeneous binder distribution and drives PVDF crystallization toward the *α*‐phase [25]. A more uniform binder dispersion reinforces mechanical cohesion and yields a cohesive, continuous, and electronically conductive CBD network in AFPA‐based electrodes. The structurally integrated CBD enabled by AFPA provides more continuous electron pathways to the current collector, which is expected to alleviate electronic bottlenecks during cell operation. Beyond electronic connectivity, X‐ray computed tomography (X‐ray CT) reveals that the AFPA‐based electrode features an open, well‐connected pore network. This highly ordered and more periodic architecture facilitates efficient Li‐ion transport. In comparison, the HNBR‐based electrode exhibits an irregular, randomly distributed pore network with localized pore clogging, which can block Li‐ion pathways and limit ionic diffusion. Pore architecture differences are closely tied to binder distribution. A pronounced binder gradient develops across the electrode thickness in the HNBR‐based electrode, whereas the AFPA‐based electrode achieves more uniform binder distribution throughout the structure. This contrast in binder distribution is strongly influenced by PVDF compatibility by drying. In HNBR‐based electrodes, PVDF tends to form more polar *β*‐phase due to limited miscibility with the binder and solvent environment, whereas in AFPA‐based systems, the favorable compatibility with both PVDF and NMP stabilizes the non‐polar *α*‐phase. The phase behavior of PVDF appears to be governed by dispersant chemistry, which in turn affects binder dispersion and structural uniformity [22]. The AFPA enables the formation of a cohesive and interconnected CBD, allowing operation at a low CNT content of 0.425 wt% without compromising electrochemical performance. This enhancement is further supported by the distinct rheological behavior observed in the CNT–dispersion and corresponding slurries, as shown in Figure S2. Relative to HNBR, AFPA strengthens CNT interactions while improving compatibility with PVDF. This helps maintain dispersion integrity during slurry mixing, suppressing re‐agglomeration and supporting a more uniform CBD.

### 2.2 Spatial Mapping of Electron Transport and CNT‐Binder Distribution in CBDs

**FIGURE 2:**
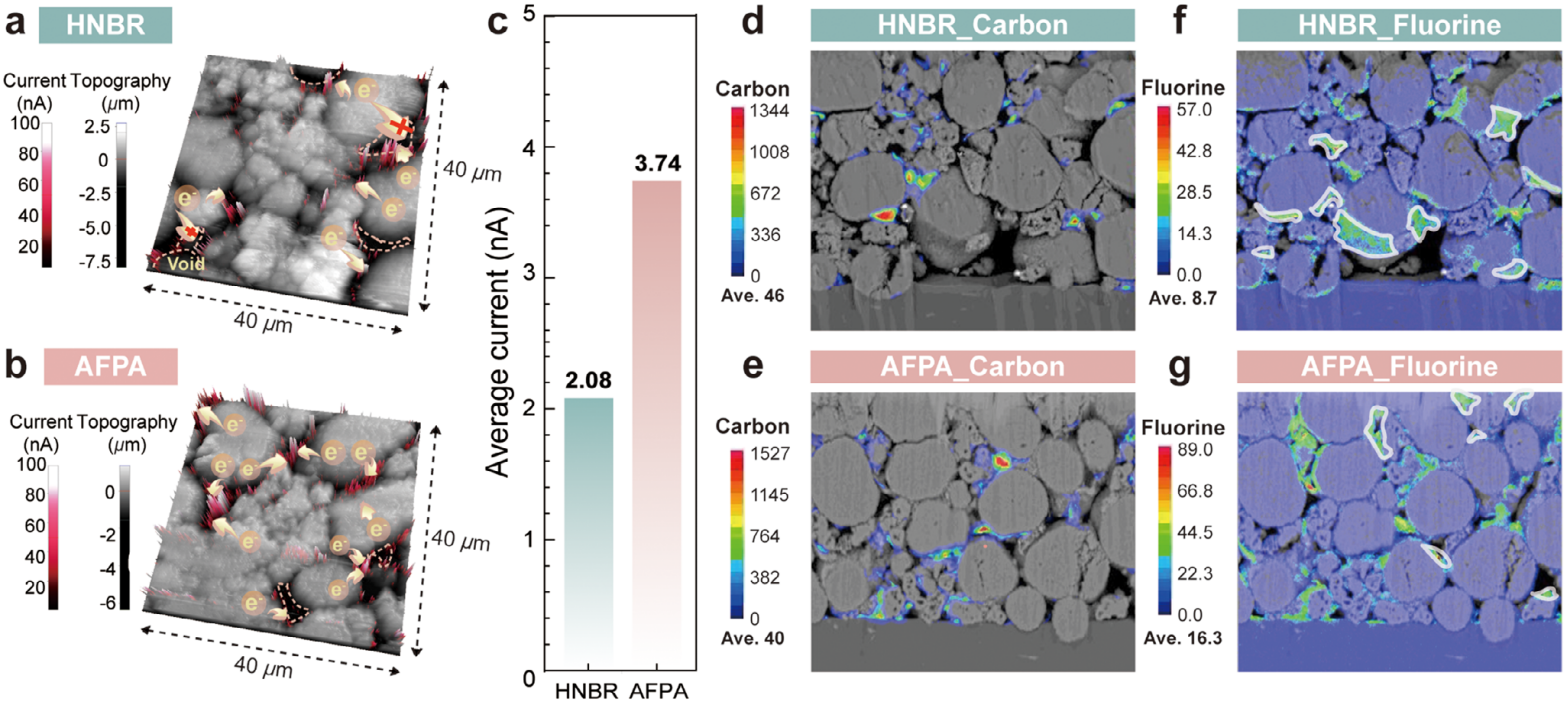
Spatial analysis of electron transport pathways as governed by dispersant‐induced CBD morphology. C‐AFM surface topography and current images of NCM811‐based electrodes prepared with (a) HNBR and (b) AFPA dispersants, with overlaid electron transport pathways and void highlighted for clarity. (c) Average current values extracted from the corresponding current images. EPMA elemental maps of carbon (d,e) and fluorine (f,g) reveal differences in the distribution of CNT and binder components between the two electrodes. White boundaries highlight regions where fluorine is detected in the absence of carbon signals.

The current distributions (magenta color) on the surface, observed through conductive atomic force microscopy (c‐AFM), were compared in Figure 2a,b. In the HNBR‐based electrode, the current signals are highly localized, indicating a fragmented conductive network that limits efficient electron transport. Additionally, large voids are observed between NCM811 particles, which may further disrupt electron pathways. In contrast, the AFPA‐based electrode exhibited a more uniform current distribution wrapped around the NCM811 particles, with fewer voids, ensuring an interconnected conductive network. To further investigate electron transport pathways, the CNT network in the electrode was analyzed in Figure S3a,b. Binder aggregation is partially evident in the HNBR‐based electrode, which restricts electron flow. As a result, electron transport in the HNBR electrode may rely on short‐range hopping between isolated NCM811 cathode particles, due to the disrupted conductive network [26]. In contrast, the AFPA‐based electrode reveals a well‐connected CNT network embedded within the CBD, providing extended pathways for direct and continuous electron transport. This robust network facilitates fast long‐range conduction across the electrode [27]. The superior electron transport efficiency of the AFPA‐based electrode is reflected in its average current measurement of 3.74 nA, significantly exceeding the 2.08 nA measured for the HNBR‐based electrode (Figure 2c).

Electron probe X‐ray microanalysis (EPMA) was conducted to examine whether the CBD distribution across the electrode cross‐section aligns with the surface characteristics, as shown in Figure 2d–g. In the HNBR‐based electrode, carbon was primarily distributed around smaller NCM811 particles, while localization at the contact points between larger particles was also observed. In contrast, in the AFPA‐based electrode, carbon was more broadly distributed around both small and large NCM811 particles, indicating a more continuous CBD network. To further investigate whether this carbon distribution correlates with PVDF binder or exists separately, the fluorine distribution was analyzed. The white outlines indicate regions where fluorine is segregated from carbon, revealing that HNBR‐based electrode exhibits broader fluorine‐rich regions. This suggests that limited compatibility between PVDF and HNBR may have led to partial phase separation. In line with this observation, optical microscopy of the CBD films showed more uniform CNT dispersion in the AFPA‐based composite, whereas the HNBR‐based composite exhibited significant CNT agglomeration (Figure S4a,b). Raman spectroscopy of the same CBD films further revealed a more pronounced G‐band blue shift in the AFPA‐based composite, indicating stronger interaction between CNTs and PVDF [28]. In the electrode‐level spectra, this difference became less apparent because the low CBD fraction (∼2.1 wt%). The AFPA‐based electrode still showed a more resolved the G’/2D‐band feature, consistent with a partial modification of the local CNT environment within the practical composite electrodes (Figure S4c,d) [29].

The electrode resistance measurement system was employed to analyze the electrical conductivity over a relatively wide range (Figure S5). Analysis of the cathode material layer and the interfacial layer resistance revealed that the AFPA‐based electrode exhibited lower resistance values compared to its counterpart. This finding is consistent with Figure 2e, which shows that CNTs in the AFPA‐based electrode form a more uniform and well‐integrated conductive network throughout the electrode. Consistently, X‐ray photoelectron spectroscopy (XPS) analysis revealed a more carbonaceous CBD‐related composition near the NCM surface in the AFPA‐based electrode, supporting effective short‐range electronic contact at the active‐material interface (Figure S6) [30]. This homogeneous distribution not only improves electron transport between the cathode materials but also ensures effective electron transfer to the current collector, thereby reducing interfacial resistance.

### 2.3 Pore‐Scale Architecture and Ionic Transport Governed by CBD Networks

**FIGURE 3:**
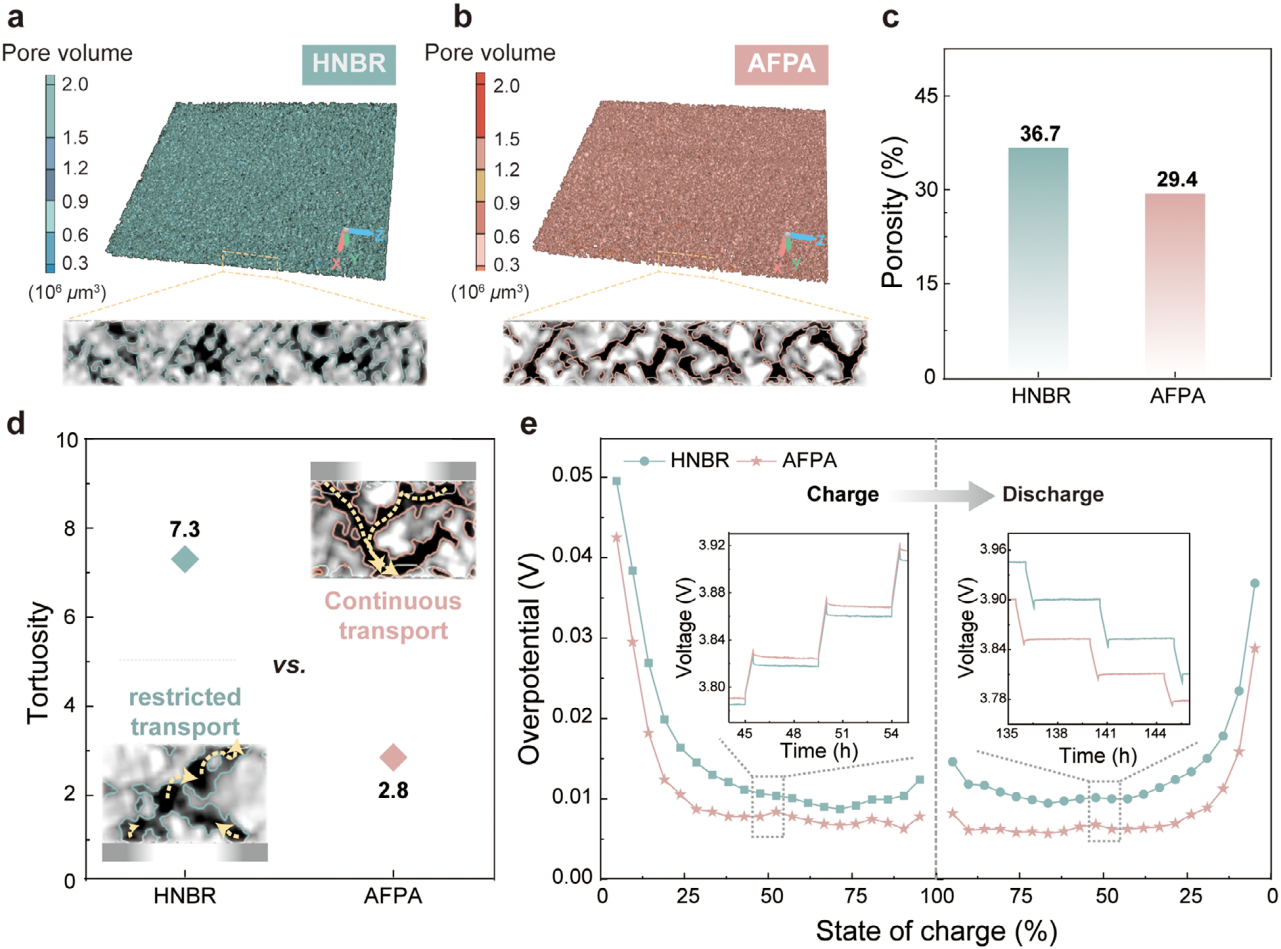
Dispersant‐driven CBD networks modulate pore architecture and ionic transport. Representative 3D X‐ray CT reconstructions of pore structures in (a) HNBR‐based and (b) AFPA‐based electrodes, with cross‐sectional views shown below each rendering. (c) Porosity extracted from X‐ray CT images. (d) Tortuosity determined by EIS, with the inset showing an X‐ray CT cross‐section used to visualize Li‐ion transport pathways. (e) Overpotential profiles from GITT with the inset presenting representative voltage–time profiles near 50% SOC during both charging and discharging steps.

Along with its role on electron transport, the distribution of the CBD plays a critical role in determining the pore structure of the electrode, which influences the ion diffusion pathway [9, 27]. X‐ray CT analysis, shown in Figure 3a,b, reveals distinct differences in pore morphology between the two electrodes. The HNBR‐based electrode featured randomly distributed pores of varying sizes, whereas the AFPA‐based electrode exhibited a more interconnected pore network with elongated pathways. This improved connectivity suggests that ion transport may be more efficient in the AFPA‐based electrode. The formation of a well‐connected porous network in electrode may originate from the uniform distribution of the CBD in the slurry state, as suggested by the rheological analysis (Figure S7). Compared with the HNBR‐based CBD solution, the AFPA‐based system exhibited a steeper shear‐thinning behavior, which is indicative of improved dispersion and structural uniformity. Notably, this enhanced uniformity is not accompanied by higher porosity, with X‐ray CT yielding 36.7% for HNBR and 29.4% for AFPA (Figure 3c). This indicates that the improved ion‐transport pathways in the AFPA‐based electrode is attributed to differences in pore morphology and connectivity, rather than an increase in porosity. Scanning electron microscopy (SEM) images (Figure S8 and Table S1) reveals more elongated pores in the AFPA‐based electrode, reflected by a higher pore aspect ratio than in the HNBR‐based electrode (3.21 vs. 2.74). This morphology is suggestive of more continuous pore pathways despite similar pore size distribution. Complementary Brunauer–Emmett–Teller (BET) analysis and mercury intrusion porosimetry (MIP) measurements were performed to further characterize the electrode pore structure (Figure S9). The AFPA‐based electrode exhibited a higher BET specific surface area than the HNBR‐based electrode, indicating a larger gas‐accessible internal surface. Although X‐ray CT showed a lower geometric porosity for the AFPA‐based electrode, MIP revealed a slightly higher porosity of 19.20% compared with 18.27% for the HNBR‐based electrode. The AFPA‐based electrode also showed a larger modal pore diameter of 0.92 µm, compared with 0.81 µm for the HNBR‐based electrode. These differences reflect the distinct pore characteristics captured by the complementary techniques [31, 32]. Together, the results suggest that a more uniform CBD distribution is associated with accessible internal surface area and characteristic pore‐throat dimensions without necessarily increasing the total geometric pore volume.

Pore connectivity relevant to ion transport can be quantified in terms of tortuosity, which describes the deviation of ion transport pathways from a straight trajectory. Tortuosity is empirically related to the MacMullin number (*N*_m_) and porosity (*ε*) as follows:

$$\tau =N_{m}/\varepsilon$$

where a higher *τ* corresponds to more tortuous ion diffusion pathways, leading to increased transport resistance [33, 34]. The AFPA‐based electrode exhibited a significantly lower *τ* of 2.8 compared to 7.3 for HNBR‐based electrode, indicating that ions in the AFPA‐based electrode follow more direct diffusion paths with reduced transport resistance (Figure 3d). Further details on the electrochemical impedance spectroscopy (EIS)‐based extraction of the MacMullin number and the resulting tortuosity are provided in Figure S10. The inset images qualitatively illustrate the tortuosity contrast, showing more restricted transport pathways in the HNBR‐based electrode and more continuous, well‐connected pathways in the AFPA‐based electrode.

To further assess how the tortuosity differences translate into electrochemical transport limitation, galvanostatic intermittent titration technique (GITT) was performed to probe diffusion‐related overpotentials (Figure 3e). The AFPA‐based electrode consistently exhibited lower overpotentials than the HNBR‐based electrode during both charging and discharging, in agreement with the symmetric‐cell EIS results (Figure S11). The larger overpotential difference during discharge suggests that ion transport is more strongly influenced by pore connectivity in this process [35]. During discharge, making pore connectivity a dominant factor in minimizing diffusion resistance and ensuring uniform ion transport. A well‐connected pore network mitigates concentration gradients and diffusion resistance, improving the efficiency of Li‐ion transport. These findings highlight a uniform CBD establishes pore connectivity and improves electrochemical performance.

### 2.4 Mechanical Properties and PVDF Phase Evolution in CBDs

**FIGURE 4:**
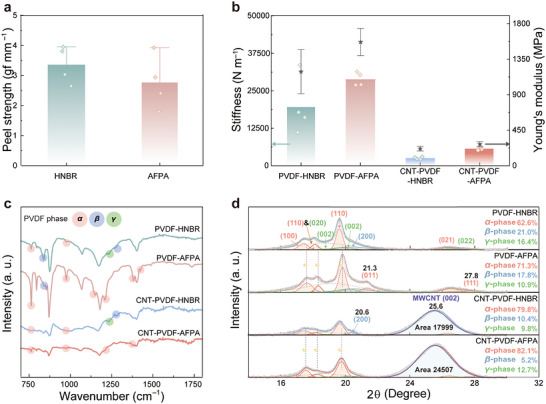
Mechanical characterization of CBD in electrodes, in relation to dispersant‐driven PVDF crystalline phase. (a) Peel strength of electrodes prepared with HNBR and AFPA. (b) Stiffness and Young's modulus of binder–dispersants and CBD composite films. The following analyses were performed to identify phase differences in PVDF, as shown in (c) FT‐IR spectra and (d) XRD profiles.

Maintaining a well‐structured electrode is essential for ensuring mechanical stability and electrochemical performance. The strong adhesion prevents electrode delamination and mechanical degradation, which are critical for maintaining structural integrity during cycling [36]. The 90° peel test confirmed that the AFPA‐based electrode exhibited a slightly lower adhesion strength of 0.7 gf mm^−1^ compared to the HNBR‐based electrode (Figure 4a). After the peeling, the AFPA‐based electrode exhibited a layered detachment pattern, whereas the HNBR‐based electrode delaminated randomly from the aluminum current collector (Figure S12). The observed differences in post‐peel morphology indicate different fracture pathways within the electrode structures [36, 37].

Despite using the same binder, these differences in failure behavior likely arise from the distinct interaction between PVDF and the two dispersants.

Universal testing machine (UTM) measurements of PVDF‐dispersant and CBD films demonstrated consistently higher Young's modulus and stiffness with AFPA‐based films than with HNBR‐based films (Figure 4b). The AFPA‐based films exhibited a 1.3‐fold higher Young's modulus than HNBR‐based films in both PVDF‐dispersant and CBD films, while stiffness increased by 1.4‐fold in PVDF‐dispersant films and nearly twofold in CBD films. The larger stiffness contrast in CNT‐containing CBD films reflects the sensitivity of stiffness to CNT network connectivity and interfacial load transfer, which AFPA better reinforces [38]. Consistent with these film‐level mechanical properties, additional measurements on high‐loading electrodes showed comparable adhesion for the two electrodes, whereas the AFPA‐based electrode exhibited a higher average cohesive strength than the HNBR‐based electrode (Figure S13). These results indicate that the mechanically reinforced AFPA‐based CBD better preserves intra‐layer integrity under high‐loading conditions.

This trend is further supported by rheological analysis (Figure S14), where AFPA‐based CBD exhibited a solid‐like response (*G´* > *G˝*) across all frequencies. In line with these result, EPMA fluorine depth profiling shows a more uniform through‐thickness binder distribution in the AFPA‐based electrode than in the HNBR‐based electrode (Figure 2d–g; Figure S15). This uniform binder profile supports a more integrated CBD for the AFPA‐based electrode and indicates stronger PVDF–dispersant interactions that suppress binder migration [39].

To determine whether the cohesion difference originates from interaction between PVDF and dispersant, the PVDF crystalline phase was analyzed using Fourier transform infrared spectroscopy (FTIR), as shown in Figure 4c. The FTIR analysis revealed that PVDF–HNBR contained a mixture of *α*‐, *β*‐, and *γ*‐phases, whereas PVDF–AFPA predominantly stabilized the *α*‐phase [40, 41]. This difference is attributed to the differing miscibility of PVDF with the dispersants: AFPA exhibits stronger interactions with PVDF, restricting molecular mobility and promoting *α*‐phase stability, while the limited compatibility between PVDF and HNBR leads to phase heterogeneity, favoring the formation of *β*‐ and *γ*‐phases [21, 22]. The relative *β*‐phase fraction, *F*(*β*), estimated from the FTIR absorbance intensities at 762 and 839 cm^−1^, was higher in the HNBR‐based films than in the corresponding AFPA‐based films (Figure S16) [42, 43]. This can be explained by localized phase separation during slurry processing in the HNBR‐based system, which promotes chain alignment and nucleates *β*‐phase domains [21, 44]. In contrast, the strong compatibility between AFPA and PVDF suppresses local chain ordering during solvent evaporation, thereby inhibiting *β*‐phase nucleation and stabilizing the non‐polar *α*‐phase structure [22, 25, 44]. Upon CNT incorporation, the AFPA‐based system retained *α*‐phase integrity, whereas the HNBR‐based system exhibited a reduction in *α*‐phase peak intensity, suggesting partial phase disruption [45].

The phase differences identified by FTIR were further confirmed by X‐ray diffraction (XRD) analysis, as shown in Figure 4d. All samples primarily exhibited *α*‐phase as the dominant crystalline phase, while the *β‐* and *γ*‐phases contents were higher in HNBR‐based films regardless of CNT presence. In comparison, AFPA‐based films exhibited a correspondingly higher *α*‐phase fraction, consistent with a shift in crystalline phase composition toward non‐polar structures [46, 47]. The phase fraction shown in Figure 4d are also in line with the *F*(*β*) values summarized in Figure S16. In particular, PVDF–AFPA was the only sample to clearly display the *α*‐phase features, with distinct *α* (011) and *α* (111) peaks at 21.3° and 27.8°, respectively, indicating a well‐defined *α*‐phase structure. This suggests that AFPA suppresses the formation of polar crystalline phases while stabilizing the *α*‐phase, due to its improved compatibility with PVDF. The *α*‐phase peak in HNBR‐based films shifted toward a lower angle than AFPA‐based films, suggesting an expansion of the PVDF d‐spacing, likely influenced by structural modifications in the crystalline arrangement. These results are consistent with previous studies that observed peak shifts corresponding to increase in *β*‐phase content [48]. Consistent with this structural relaxation, differential scanning calorimetry (DSC) analysis revealed a decrease in both crystallization temperature (*Tc*) and melting point (*Tm*) compared to AFPA‐Based films (Figure S17). This suggests that weakened intermolecular interactions and reduced crystalline stability may compromise the cohesive strength and structural integrity within the CBD [49].

Upon CNT incorporation, the (002) peak appeared at 25.6°, accompanied by an increase in *α*‐phase content [46]. Even so, the *β* (200) peak at 20.6° remained twice as high in HNBR‐based CBD compared to AFPA‐based one, suggesting that *β*‐phase crystallization was not fully suppressed in the HNBR‐based system. Furthermore, the (002) peak intensity in AFPA‐based CBD was 1.3 times higher than in HNBR‐based CBD, indicating a greater degree of CNT alignment [50]. Such alignment facilitates the formation of continuous and well‐connected conductive networks, which enhance electron mobility across the CBD, highlighting the importance of nanoscale structural organization in optimizing electrode‐level conductivity.

### 2.5 Dispersant–PVDF Interactions and Phase Behavior From MD Simulation

**FIGURE 5:**
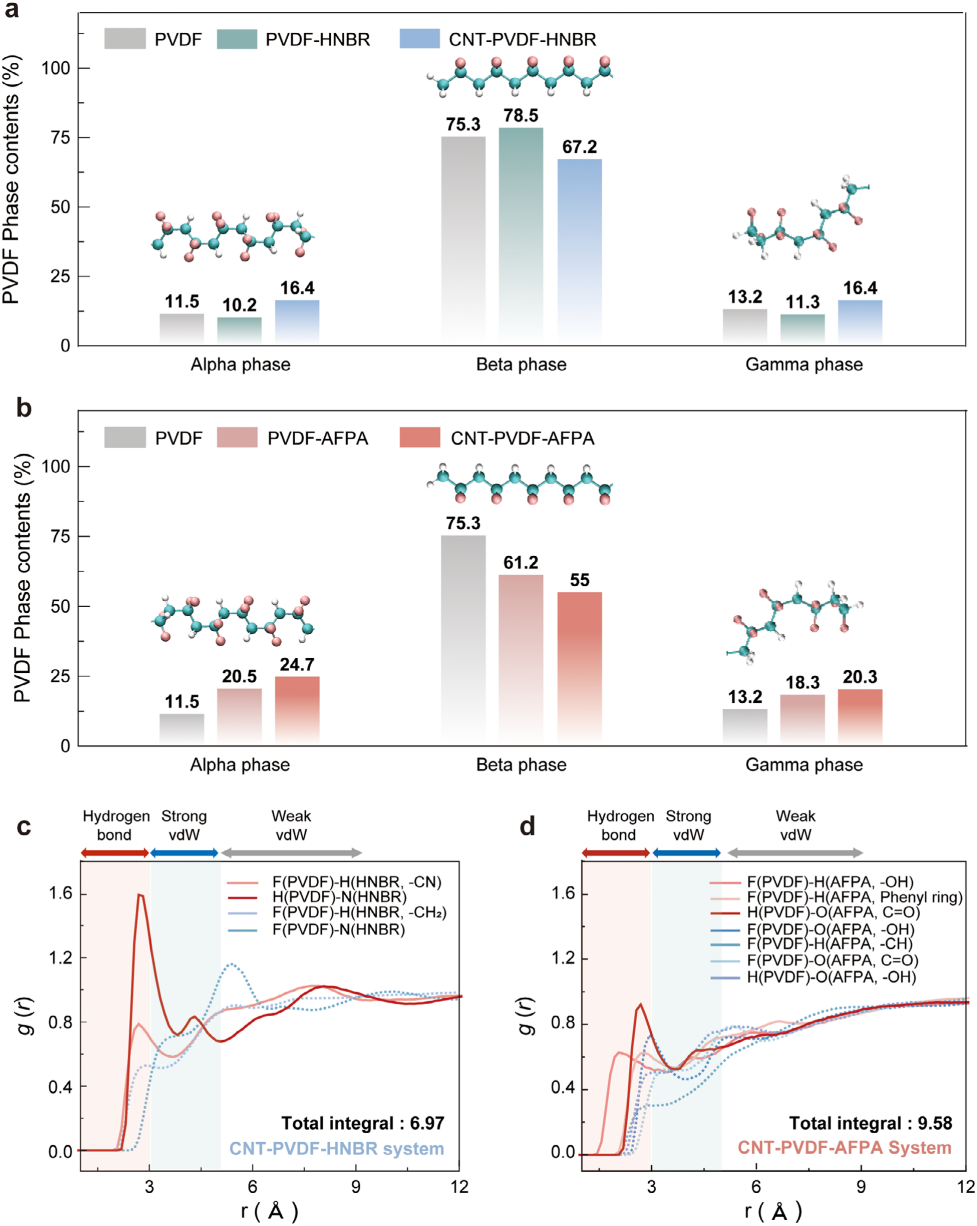
MD simulation of PVDF phase distribution and its molecular interaction with dispersants in binder and CBD systems. Phase contents of PVDF calculated from MD simulations for pristine PVDF, PVDF with dispersant, and CNT–PVDF with dispersant, in the HNBR (a) and the AFPA (b) systems. Inset molecular structures represent the *α*, *β*, and *γ* conformations of PVDF phase. RDF profiles showing interactions between PVDF functional groups and dispersant functional groups in CNT‐PVDF‐HNBR (c) and CNT‐PVDF‐AFPA (d).

To further investigate the PVDF phase behavior observed in Figure 4, molecular dynamics (MD) simulations were performed under initial drying conditions at 80°C. The *α*‐phase predominates in the experiments (Figure S18), which is consistent with its thermodynamic stability [51]. In contrast, our MD simulations predict a predominant of the *β*‐phase in PVDF. This discrepancy is likely due to the idealized MD crystal model not accounting for the complexities of real materials, such as chain folding [52]. Figure 5a,b presents the simulated phase composition of PVDF films with different dispersants. Neat PVDF consisted of 75.3% *β*‐phase, 11.5% *α*‐phase, and 13.2% *γ*‐phase. The addition of HNBR resulted in a slight increase in the *β*‐phase content (∼3.2%) with marginal reductions in *α*‐ and *γ*‐phases. In contrast, the introduction of AFPA induced a substantial phase shift with a 14.1% reduction in the *β*‐phase and corresponding increases of 9.0% and 5.1% in the *α*‐ and *γ*‐phases, respectively. This notable phase transition suggests stronger interaction between AFPA and PVDF, which likely contribute to *α*‐phase stabilization, as further confirmed in Figure S19. When comparing PVDF phase evolution as a function of dispersant molecule count, HNBR showed negligible influence on the phase composition, regardless of the number of dispersant molecules. In contrast, increasing the number of AFPA molecules led to a gradual decrease in the *β*‐phase and accompanied by corresponding increases in the *α*‐ and *γ*‐phases. These results are attributed to the differences in miscibility between PVDF and the respective dispersant molecules [22, 44]. Upon CNT introduction, both *α*‐ and *γ*‐phases were found to increase, while the *β*‐phase decreased, regardless for both types of dispersant molecules. This suggests that in the presence of dispersants, CNTs are uniformly dispersed within the PVDF matrix, promoting strong π–π interactions that help stabilize the *α*‐phase as the dominant crystalline structure [53].

Radial distribution functions (RDFs, *g*(*r*)) were computed to examine molecular‐level interactions between functional groups of dispersants and PVDF chains within the CBD system (Figure 5c,d). The RDF describes the probability of finding functional groups of dispersants at specific distances from functional groups of PVDF chains, providing insight into spatial organization and interaction strength. The AFPA‐based CBD with more functional groups capable of interacting with PVDF, exhibited a higher total integration value of 9.58, compared to 6.97 of HNBR‐based CBD. The first peak in the RDF represents the most probable atomic pair distance, providing insights into the spatial distribution and strength of intermolecular interaction [54]. For AFPA, the first RDF peak appears at 2.0 Å, corresponding to strong hydrogen bonding between hydroxyl (─OH) groups and PVDF fluorine. In contrast, the primary peak in HNBR emerges at 2.7 Å, attributed to dipole interactions between cyano (─CN) groups and PVDF hydrogen [55]. Although this distance falls within the conventional hydrogen bonding range, the absence of a direct hydrogen donor–acceptor interaction suggests a predominantly dipolar interaction [56]. Beyond these short‐range interactions, the 3–5 Å region corresponds to strong van der Waals forces [51]. In this range, AFPA maintains *g*(*r*) values near unity, indicating a uniform distribution of interactions across the network. In contrast, HNBR shows a sharp peak (∼1.6) at short range, followed by a steep decline, suggesting strong but localized interactions. These findings indicate that the AFPA‐based CBD promotes a more evenly distributed interaction network, which may suppress localized aggregation and improve phase stability within the system [57].

A comparison with the PVDF–dispersant systems offers additional insight into the structural response of each dispersant upon CNT incorporation. As shown in Figure S20 and S21, the AFPA‐based CBD undergoes minimal reorganization upon CNT addition and remains primarily associated with PVDF. By contrast, the HNBR‐based CBD redistributes toward the CNT, indicating preferential partitioning of HNBR at the CNT interface and CNT‐centered reorganization of the network. The combined Figures 4 and 5 demonstrate that interactions among CBD components drive PVDF phase transitions, which in turn contribute to electrode stabilization and structural integrity.

### 2.6 Digital Twin Prediction of Dispersant‐Dependent CBD Structure at High Mass Loading

**FIGURE 6:**
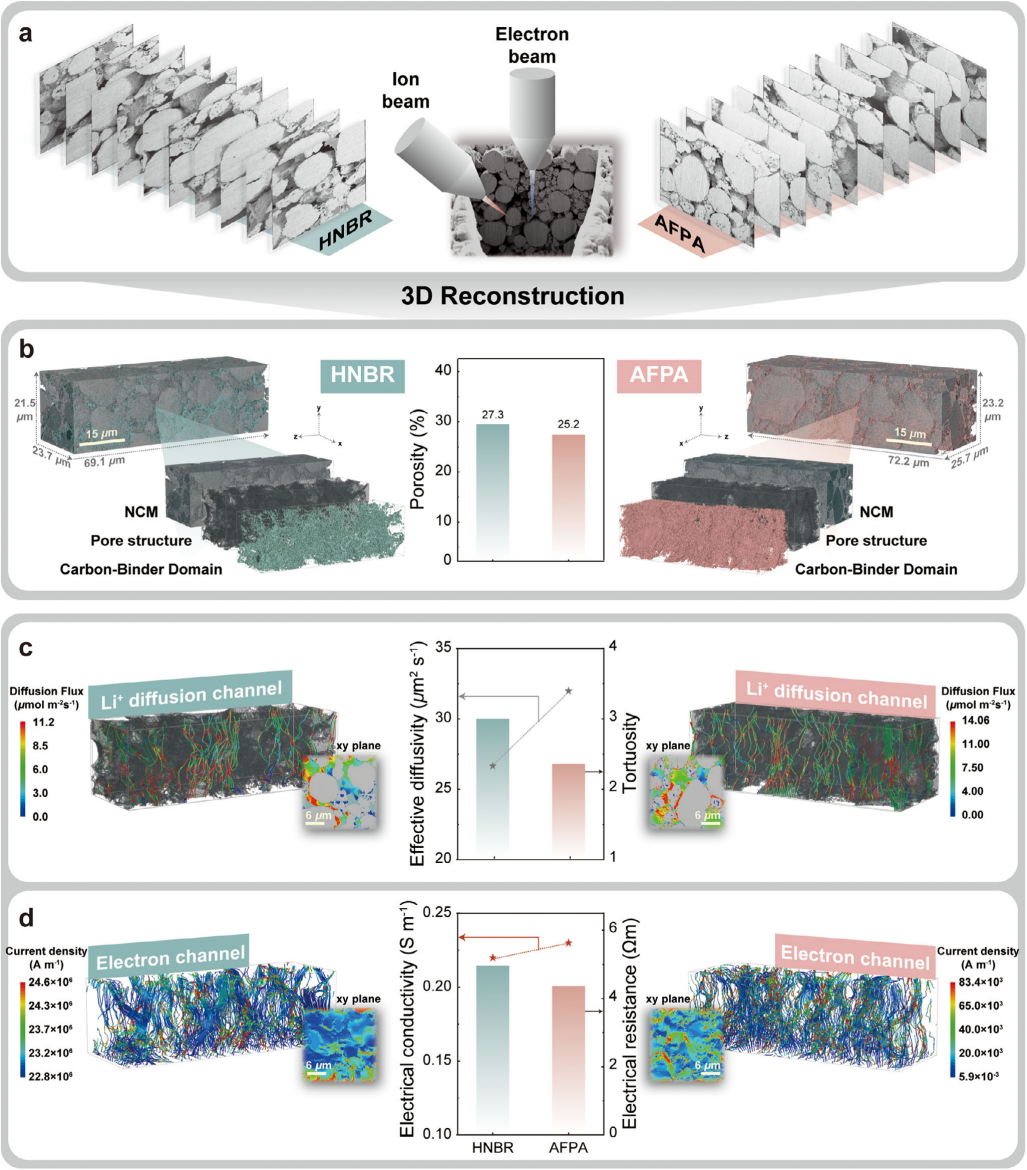
3D reconstruction and digital twin mapping of CBD microstructure in HNBR‐ and AFPA‐based electrodes at 20 mg cm^−2^. (a) FIB‐SEM tomography for 3D reconstruction. (b) 3D segmentation of the reconstructed electrodes and porosity quantification. (c) Effective ionic diffusivity and tortuosity with Li‐ion diffusion channel visualization. (d) Electrical conductivity and resistance with electron channel visualization.

Building on the microstructural trends identified at 10 mg cm^−2^, an image‐based digital twin was constructed for an electrode at 20 mg cm^−2^ to probe ionic and electronic transport at practically relevant mass loading. The 3D microstructure was reconstructed from approximately 400 focused ion beam‐scanning electron microscopy (FIB‐SEM) images, followed by image processing and segmentation (Figure 6a). Three‐phase segmentation of the reconstructed electrodes into the active material, pore structure, and CBD is shown in Figure 6b. The HNBR electrode exhibited a porosity of 27.3%, whereas the AFPA electrode showed 25.2%, potentially reflecting differences in CBD distribution and following the trend observed by X‐ray CT (Figure 3c).

Lower porosity in the AFPA electrode was accompanied by a higher effective ionic diffusivity (D_eff_, 32 vs. 27.3 um^2^ s^−1^) in the pore structure, which is consistent with its reduced effective Li^+^ diffusion tortuosity (*τ*_diff_) and more uniformly distributed diffusion pathways (Figure 6b,c) [58]. Indeed, the *τ*_diff_ was lower for the AFPA electrode by 0.64 than for the HNBR electrode (2.36 vs. 3.00). In contrast, geometric tortuosity (*τ*_geo_) was comparable (Figure S22). In short, *τ*_geo_ represents only the paths that can be taken in the 3D electrode model, and *τ*_diff_ represents whether the path is one in which Li^+^ can flow well in the 3D electrode model. From the results, it is inferred that a fine difference in *τ*_geo_ will lead to a larger difference in *τ*_diff_ in terms of actual Li^+^ diffusion. Notably, the xy‐plane Li‐ion concentration map showed greater spatial heterogeneity in the HNBR electrode with distinct low‐concentration regions (blue), whereas the AFPA electrode displayed a more uniform distribution, in agreement with the ionic‐transport trends in Figure 3 [23].

Along with ionic transport, electronic transport was also evaluated to assess how the dispersant‐assisted CNT network influenced current distribution in the high‐loading electrodes (Figure 6d). Relative to the HNBR electrode, the AFPA electrode showed only minor differences in bulk electronic transport, with the electronic conductivity increasing by 3.3% (0.22 to 0.23 S m^−1^) and the electronic resistivity decreasing by 12% (4.96 to 4.37 Ω m). Despite these limited bulk differences, the current‐density maps revealed distinct pathway distributions. The HNBR electrode showed more locally clustered channels, and the AFPA electrode exhibited a more spatially distributed network. In the HNBR electrode, high current density (red) was more localized near the top and bottom regions, whereas the AFPA electrode showed a more uniform distribution around the active material across the xy plane [59, 60].

These transport trends were further corroborated by experimental measurements of electronic conductivity, ionic conductivity, and electrolyte uptake at the same loading condition (Figure S23). The AFPA‐based electrode exhibited higher values for all three properties than the HNBR‐based electrode, consistent with the digital‐twin results showing higher effective diffusivity, lower tortuosity, and improved electrical transport. Together, the digital‐twin and experimental transport analyses clarify the structure–transport relationship at high mass loading, revealing more localized transport pathways in the HNBR‐based electrode and more spatially distributed pathways in the AFPA‐based electrode. This result indicates that dispersant‐driven differences in CBD uniformity remain evident even under high mass loading.

### 2.7 Electrochemical Performance of CBD Uniformity at Reduced CNT Loading

**FIGURE 7:**
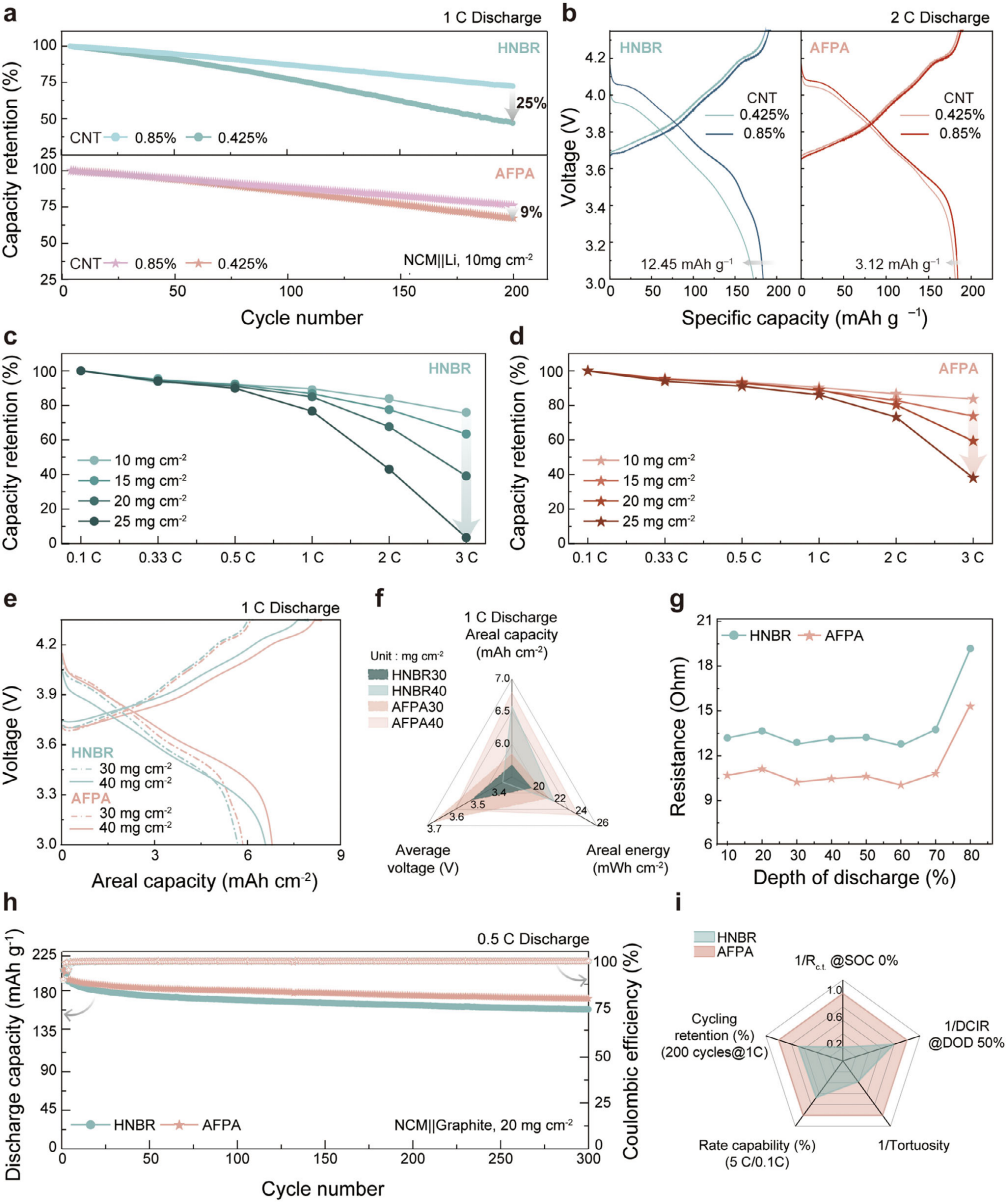
Electrochemical performance of high‐loading NCM811 electrodes with different dispersants. (a,b) NCM811‖Li half cells with two CNT contents (0.425 and 0.85 wt%) using HNBR or AFPA dispersants. (a) Capacity retention during long‐term cycling at 1 C. (b) Voltage profiles at 2 C from rate test. (c,d) Rate capability of HNBR and AFPA‐based electrode from 0.1 to 3 C as a function of mass loading. (e) 1 C voltage profiles from rate tests at high mass loading (30 and 40 mg cm^−2^). (f) Radar‐plot summary of areal capacity, average voltage, and areal energy extracted from (e). (g) DCIR comparison as a function of DoD. (h) NCM811‖Graphite pouch‐cell cycling performance at 20 mg cm^−2^. (i) Summary plot linking CBD microstructure to electrochemical performance across the dataset at 10 mg cm^−2^. All electrochemical measurements were performed at room temperature.

Since the CBD microstructure governs electronic connectivity, ionic pathways, and mechanical integrity, we assessed how CBD differences between HNBR‐ and AFPA‐based electrodes manifest in electrochemical performance, with negligible contribution from CNT‐derived metal residues (Figure S24). Decreasing the conductive additive fraction toward the percolation threshold limits the ability to sustain a percolating electronic network [61, 62]. This stronger dependence on CBD homogeneity becomes evident when the CNT content is reduced by 50% from 0.85 to 0.425 wt%. The HNBR electrode exhibited a 25% loss in capacity retention after 200 cycles, compared with a 9% loss for the AFPA electrode (Figure 7a; Figure S25). Under low‐CNT conditions, the accelerated capacity loss observed for the HNBR electrode was consistent with increased cycling‐induced structural degradation [30, 61].

At 2 C, polarization was more pronounced, and reducing the CNT content further decreased the discharge capacity. When CNT content was reduced by 50%, the discharge capacity drop was 12.45 mAh g^−^^1^ for HNBR, compared with 3.12 mAh g^−^^1^ for AFPA (Figure 7b; Figure S25). These results indicate that forming a uniform CBD at reduced conductive additive fractions is essential for maintaining continuous electronic connectivity, supporting high‐energy‐density electrode designs.

Beyond composition, high‐energy‐density electrode designs often involve higher active material loading, which thickens the electrode and amplifies transport limitations through the thickness direction [62, 63]. These limitations become more sensitive to CBD homogeneity because the CBD microstructure modulates both electronic connectivity and pore structure for ion transport [23, 62]. In Figure 7c,d, capacity retention at c‐rates up to 3 C was defined relative to the 0.1 C capacity as the active material loading increased from 10 to 25 mg cm^−^^2^. Higher active material loading intensified the c‐rate dependent loss in capacity retention, and the difference between the two electrodes became clearer at 2 C and above. The most evident contrast was seen at 3 C, where capacity retention was reduced by 95.4% for the HNBR electrode (from 74.74% to 3.45%) when the active material loading was increased from 10 to 25 mg cm^−^^2^, compared to the 55.0% reduction for the AFPA electrode (from 84.53 to 46.51%). This trend was consistent with the experimentally observed CBD uniformity at 10 mg cm^−^^2^ in Figures 2 and 3 and further aligned with the digital twin analysis based on 20 mg cm^−^^2^ in Figure 6.

Moreover, the mass loading range was extended to 30 and 40 mg cm^−^^2^, as reflected in 1 C discharge profiles in Figure 7e. Despite similar areal capacities of 5.5–7.0 mAh cm^−^^2^, the HNBR electrode exhibited a larger initial IR drop and greater polarization, resulting in a lower discharge voltage [62, 63]. A lower average discharge voltage, especially at 40 mg cm^−^^2^, resulted in a lower areal energy while the areal capacity remained comparable. To facilitate comparison at higher loading, we summarized areal capacity, average voltage, and areal energy extracted from the 1 C discharge profiles as radar plots (Figure 7f). The larger discharge polarization observed for the HNBR electrode was corroborated by the direct current internal resistance (DCIR) results, which remained higher than those of the AFPA electrode across the entire depth of discharge (DoD) range (Figure 7g). The EIS spectra further showed that the Nyquist profiles were initially similar but became clearly different after cycling, with the HNBR electrode exhibiting a more pronounced increase in apparent interfacial resistance and polarization than the AFPA electrode (Figure S26) [64]. These results underscore that the more homogeneous CBD enabled by AFPA electrode mitigates internal resistance growth and discharge polarization, leading to higher areal energy at high loading [65, 66, 67].

Full‐cell cycling at 20 mg cm^−2^ also indicates that the AFPA‐based electrode retains higher discharge capacity over 300 cycles at 0.5 C (Figure 7h). A lower discharge capacity of the HNBR electrode in the initial cycles may reflect its higher polarization, which can accelerate reaching the cut‐off voltage and limit active‐material utilization. This behavior was further assessed under more practically relevant full‐cell conditions using NCM811‖SiC full cells with a higher cathode loading of 30 mg cm^−2^ and controlled electrolyte‐to‐capacity (E/C) ratios (Figure S27). At an E/C ratio of 3.0 g Ah^−1^, the pouch‐cell configuration corresponded to calculated cell‐level gravimetric and volumetric energy densities of 252.3 Wh kg^−1^ and 867.5 Wh L^−1^, respectively (Figure S27 and Tables S2 and S3) [68]. Both cells initially showed stable cycling at 0.33 C with only a modest difference in discharge capacity, although prolonged cycling revealed an earlier onset of accelerated capacity fading in the HNBR‐based cell. The performance difference became more pronounced when the E/C ratio was reduced to 2.5 g Ah^−1^. The AFPA‐based cell maintained nearly stable capacity over 100 cycles, whereas the HNBR‐based cell showed continuous capacity decay accompanied by progressive polarization growth. Lowering the E/C ratio reduced the inactive electrolyte mass and increased the calculated cell‐level gravimetric energy density from 252.3 to 261.5 Wh kg^−1^ (Figure S27 and Table S2) [69]. Thus, the reduced‐E/C configuration provides a practical route toward higher cell‐level gravimetric energy density while imposing more demanding electrolyte‐limited conditions. These results demonstrate the importance of a homogeneous CBD architecture for maintaining capacity utilization and cycling stability in high‐loading full cells under lean‐electrolyte conditions.

The practical implications of CBD uniformity were also examined at the maximum calendaring density achieved for each electrode. At a loading of 20 mg cm^−2^, the HNBR‐ and AFPA‐based electrodes reached maximum densities of 3.5 and 3.6 g cc^−1^, respectively. Notably, the AFPA‐based electrode retained superior rate capability even at the higher degree of densification, whereas the HNBR‐based electrode showed a more pronounced loss in capacity at elevated C‐rates (Figure S28a). This behavior is consistent with a more homogeneous CBD architecture that may better sustain charge‐transport pathways under severe electrode compression [70]. The AFPA‐based electrode also showed a markedly lower degree of spring‐back, indicating better retention of the thickness reduction achieved by calendaring (Figure S28b). Together, these results suggest that a homogeneous CBD architecture helps high‐loading electrodes better withstand severe calendaring and preserve the volumetric benefit gained through densification [71].

To contextualize this cell‐level result, Figure 7i provides an overview of key structure and transport descriptors relevant to electrochemical performance at 10 mg cm^−2^, including 1/tortuosity, 1/DCIR, and 1/*R*_c.t._ along with rate capability and cycling retention (Table S4). The overall trend in favor of AFPA supports a more homogeneous CBD distribution that mitigates ionic and electronic transport limitations, resulting in improved electrochemical performance.

## 3 Conclusion

In this study, we demonstrate that compatibility among CBD constituents governs CBD homogeneity and thereby influences electrochemical performance, even at an ultralow CNT content of 0.425 wt%. The AFPA dispersant exhibits improved miscibility with PVDF in NMP and yields a more homogeneous CBD compared with the conventional HNBR dispersant. This homogeneity enables a continuous conductive network and maintains connected ion transport pathways near the percolation threshold, as confirmed by direct experiments and an image‐based digital twin.

The uniform CBD microstructure reflects PVDF crystallization behavior, with its crystallinity influencing binder distribution within the CBD and enhancing CBD film cohesion in the AFPA‐based electrode. This cohesion helps maintain electronic and ionic pathways during cycling and high‐rate operation, mitigating polarization and thereby improving rate capability and cycling stability. AFPA‐based electrodes delivered 159.6 mAh g^−1^ at 5 C and retained 67.3% after 200 cycles, whereas HNBR‐based electrodes reached 108.8 mAh g^−1^ at 5 C and maintained 46.9%. These results underscore the optimized AFPA‐based CBD, particularly its uniformity, as a key factor governing electrochemical stability.

Importantly, in thick, high‐loading electrodes, CBD heterogeneity becomes increasingly detrimental to electrochemical performance. In particular, the heterogeneous CBD formed with HNBR exacerbates polarization at mass loadings of 30–40 mg cm^−2^, lowering the average discharge voltage and thereby reducing both areal capacity and, more markedly, areal energy.

These findings underscore that molecular‐level interactions among CBD components, particularly CNTs, PVDF, and the dispersant, govern CBD microstructure and the resulting ion and electron transport pathways. Accordingly, optimizing inter‐component compatibility within the CBD emerges as a critical engineering strategy to advance high‐energy LIBs, especially in high‐energy‐density electrodes with reduced inactive components and in high‐loading electrodes where CBD functionality becomes more critical.

## 4 Experimental Section

### 4.1 Materials

HNBR was acquired from Arlanxeo High Performance Elastomers Co., Ltd. (France), and AFPA, consisting of a copolymer derivative of polyacrylic acid and styrene, was obtained by Hansol Chemical Co., Ltd. (Korea). The dispersant/multi‐walled carbon nanotubes (MWCNTs) dispersions were prepared in N–Methyl–2–pyrrolidone (NMP) by Advanced Nano Products Co., Ltd. (Korea). The MWCNTs had a purity of 96%, an average diameter of approximately 10 nm, and an average length of 16 µm. PVDF (Solef 5130, Solvay, Belgium) was dissolved in NMP to prepare a 10 wt% solution. LiNi_0.8_Co_0.1_Mn_0.1_O_2_ (NCM811), was manufactured by POSCO Chemical Co. Ltd. (Korea) and purchased from Welcos Co., Ltd. (Korea). 1 M lithium hexafluorophosphate (LiPF_6_) in mixture of ethylene carbonate (EC) and dimethyl carbonate (DMC) (1:1 vol%) and polypropylene separator (Celgrard 2400, thickness: 26 µm) were obtained from the same supplier.

### 4.2 Preparation of Electrode

The NCM811 slurry was prepared by dispersing NCM811 as active material, MWCNTs (0.425 or 0.85 wt%), PVDF binder (1.50 wt%), and either HNBR or AFPA as the dispersant (0.21 wt%). The resulting mixture had a total solid content of 74.1 wt%, and was homogenized by high‐shear mixing at 3000 rpm for 15 min at room temperature. The prepared slurry was casted onto aluminum (Al) foil (thickness: 15 µm) using a doctor blade and dried in an oven at 80°C for 2 h, and subsequently vacuum dried at 120°C for 12 h to completely remove the NMP. The electrodes were calendered to a density of 3.2 g cc^−1^, giving a calculated porosity of approximately 30–33% as estimated from the areal mass loading, electrode thickness, component weight fractions, and true densities of the electrode components. The areal mass loading of the active materials was varied from 10 to 40 mg cm^−2^, corresponding to an areal capacity of 2.1–8.4 mAh cm^−2^ based on the NCM811 specific capacity of 210 mAh g^−1^.

Graphite anodes were prepared from a water‐based slurry (solid content: 50.2 wt%) containing graphite, carboxymethyl cellulose (CMC), styrene–butadiene rubber (SBR), a carbon black (C65) dispersion, and a single‐walled carbon nanotube (SWCNT) dispersion. The slurry was mixed using a Thinky mixer (ARE‐300) at 1300 rpm for 15 min, coated onto Cu foil, and dried at 100°C for 10 h in a vacuum oven. The graphite anode composition was composed of graphite, CMC, SBR, C65, SWCNT, and a CMC‐derived polymeric dispersant at a weight ratio of 95.86:1.20:2.40:0.45:0.035:0.05. For Si/C anode was prepared using the same procedure, with a composition of Si/C (Si/Graphite = 17.65 wt%), CMC, SBR, C65, SWCNT, and CMC‐derived polymeric dispersant at a weight ratio of 95.1:1.2:3:0.45:0.1:0.15.

### 4.3 Electrochemical Characterization

The galvanostatic charge/discharge test of 2032‐coin cells was performed using a WBCS‐3000 battery cycle system (WonATech Co., Korea). The NCM811|Celgard2400|Li half‐cells were cycled within a cut‐off voltage range of 3.0–4.35 V (*vs*. Li/Li^+^) at room temperature, where 1 C corresponds to a current density of 210 mA g^−1^. After three formation cycles at 0.1 C, the coin cells were charged using a constant current/constant voltage (CC/CV) protocol, with a cut‐off voltage of 4.35 V. Discharge for cycling was performed at a constant current of 1 C, and rate performance was evaluated by discharging the cells at current rates ranging from 0.1–5 C. GITT measurements were carried out using a current pulse of 0.1 C for 30 min, followed by a rest period of 4 h. DCIR was taken at a base current of 0.33 C and a peak current of 3 C for 30 s, within a voltage range of 3–4.35 V. EIS was conducted using a ZIVE MP1 electrochemical workstation (WonATech Co., Korea) in a frequency range from 0.1 to 100 MHz with 10 mV of amplitude at room temperature, employing a symmetric cell configuration. A 1 m LiPF_6_ in EC:DEC (1:1 v/v, 200 µL) was used as a liquid electrolyte, and all the coin cell components were assembled in an argon‐filled glove box (O_2_ < 0.1 ppm, H_2_O < 0.1 ppm).

Pouch cells were assembled in a dry room using NCM811 cathodes. The cathode area was fixed at 19.04 cm^2^ for all pouch cells. The pouch cell configuration was adjusted according to the norminal cathode areal capacity, using graphite anodes for the 4 mAh cm^−2^ cathodes and Si/C anodes for the 6 mAh cm^−2^ cathodes. All pouch cells used the same carbonate‐based electrolyte, consisting of 1 m LiPF_6_ in EC/ethyl methyl carbonate (EMC) (3:7 v/v) with 2 wt% vinylene carbonate (VC) and 0.5 wt% 1,3‐propanesultone (PS). This electrolyte was used to support electrolyte wetting and interfacial stabilization in NCM811 full cells. The electrolyte amount was specified for each pouch cell condition together with the corresponding electrolyte‐to‐capacity ratio (E/C ratio). For pouch cells using 4 mAh cm^−2^ cathodes, 230 µL of electrolyte was added per cell, corresponding to an E/C ratio of 3.5 g Ah^−1^. For pouch cells using 6 mAh cm^−2^ cathodes, 250–300 µL of electrolyte was added per cell, corresponding to E/C ratios of approximately 2.5 and 3.0 g Ah^−1^, respectively. The anode areal capacity, negative‐to‐positive capacity ratio (N/P ratio), and voltage window were 4.3–4.5 mAh cm^−2^, 1.1, and 2.8–4.2 V, respectively, for the graphite anode pouch cells, and 6.5–6.6 mAh cm^−2^, 1.1, and 2.5–4.2 V, respectively, for the Si/C anode pouch cells.

To compare the influence of CNT‐derived metal residues, cyclic voltammetry (CV) and linear sweep voltammetry (LSV) were conducted using CNT‐PVDF films prepared with and without purification. CV was recorded over 3.0–4.5 V at 0.1 mV s^−1^, and LSV was recorded from 3.0 to 5.0 V at 1 mV s^−1^.

## Author Contributions

D.‐S. Kwon, M.Y. Seo, and J. Ahn contributed equally to this work. D.‐S. Kwon conducted the experiments and anlyzed the data. M.Y. Seo and Y. Kim performed the molecular dynamics simulations. J. Ahn, W. Ko, and J. Bang contributed to the structural and spectroscopic anlayses. Y.‐J. Jeong, W.‐Y Jeon and M.‐J. Choi carried out the electrochemical measurements. M.J. Kim and J. Lee assisted the ion transport‐related analyses. K.I. Kim, J.J. Eom, and S.Y. Oh developed the dispersant materials. W. Shin and H. Lee performed various simulations based on digital twin analysis using Geodict software. D.‐S. Kwon, W. Shin, Y. Kim, and J. Kim wrote the paper with contributions from all authors. J. Kim, Y. Kim, and J.‐K Yoo conceived the idea and supervised the project.

## Conflicts of Interest

The authors declare no conflicts of interest.

## Supporting information




**Supporting File**: advs77293‐sup‐0001‐SuppMat.docx.

## Data Availability

The data that support the findings of this study are available from the corresponding author upon reasonable request.

## References

[1] J. Wu , X. Zhang , Z. Ju , et al., “From Fundamental Understanding to Engineering Design of High‐Performance Thick Electrodes for Scalable Energy‐Storage Systems,” Advanced Materials 33 (2021): 2101275, 10.1002/adma.202101275. 34028911

[2] J. U. Choi , N. Voronina , Y. K. Sun , and S. T. Myung , “Recent Progress and Perspective of Advanced High‐Energy Co‐Less Ni‐Rich Cathodes for Li‐Ion Batteries: Yesterday, Today, and Tomorrow,” Advanced Energy Materials 10 (2020): 2002027, 10.1002/aenm.202002027.

[3] J.‐H. Kim , N.‐Y. Kim , Z. Ju , et al., “Upscaling High‐Areal‐Capacity Battery Electrodes,” Nature Energy 10 (2025): 295–307, 10.1038/s41560-025-01720-0.

[4] M. Du , Z.‐L. Hao , Y. Liu , et al., “Architecture Engineering for Thick Electrodes in High‐Energy Batteries: Challenges and Strategies,” ACS Applied Materials & Interfaces 17 (2025): 19230–19246, 10.1021/acsami.5c00264. 40101175

[5] W. Xie , Z. Zhang , and X. Gao , “Understanding the limitations of thick electrodes on the rate capability of high‐energy density lithium‐ion batteries,” Electrochimica Acta 493 (2024): 144396, 10.1016/j.electacta.2024.144396.

[6] X. Zhang , Z. Ju , Y. Zhu , et al., “Multiscale Understanding and Architecture Design of High Energy/Power Lithium‐Ion Battery Electrodes,” Advanced Energy Materials 11 (2020): 2000808, 10.1002/aenm.202000808.

[7] Z. Yan , L. Wang , and X. He , “Rational Electrode Design for Enhanced Battery Performance: Addressing SOC Heterogeneity and Achieving Energy Density,” Advanced Functional Materials 35 (2024): 2415637, 10.1002/adfm.202415637.

[8] W. Jin , H. Cha , S. Choi , and G. Song , “Electrode‐Level Strategies for High‐Ni Cathodes in High‐Energy‐Density Batteries Beyond Material Design,” Energy Materials 5 (2025): 500130, 10.20517/energymater.2025.57.

[9] J. Entwistle , R. Ge , K. Pardikar , R. Smith , and D. Cumming , “Carbon binder domain networks and electrical conductivity in lithium‐ion battery electrodes: A critical review,” Renewable and Sustainable Energy Reviews 166 (2022): 112624, 10.1016/j.rser.2022.112624.

[10] M. Kroll , S. L. Karstens , M. Cronau , et al., “Three‐Phase Reconstruction Reveals How the Microscopic Structure of the Carbon‐Binder Domain Affects Ion Transport in Lithium‐Ion Batteries,” Batteries & Supercaps 4 (2021): 1363–1373, 10.1002/batt.202100057.

[11] H. Hamed , S. Yari , J. D'Haen , et al., “Demystifying Charge Transport Limitations in the Porous Electrodes of Lithium‐Ion Batteries,” Advanced Energy Materials 10 (2020): 2020002492, 10.1002/aenm.202002492.

[12] T. Lombardo , A. C. Ngandjong , A. Belhcen , and A. A. Franco , “Carbon‐Binder Migration: A Three‐Dimensional Drying Model for Lithium‐ion Battery Electrodes,” Energy Storage Materials 43 (2021): 337–347, 10.1016/j.ensm.2021.09.015.

[13] I. Kim , J. H. Choi , H. Jang , et al., “Troubleshooting Carbon Nanotube Bundling Using Electrostatic Energy‐Driven Dispersion for LiFePO_4_ Bimodal Thick Electrode in Lithium‐Ion Batteries,” ACS Nano 19 (2025): 15941–15952, 10.1021/acsnano.5c01892. 40249366

[14] Y. J. Choi , E. J. C. Nacpil , J. Han , C. Zhu , I. S. Kim , and I. Jeon , “Recent Advances in Dispersant Technology for Carbon Nanotubes Toward Energy Device Applications,” Advanced Energy and Sustainability Research 5 (2024): 2300219, 10.1002/aesr.202300219.

[15] J. Hoon Kim , J. Ahn , H.‐M. Kim , et al., “Highly Efficient Oxidation of Single‐Walled Carbon Nanotubes in Liquid Crystalline Phase and Dispersion for Applications in Li‐Ion Batteries,” Chemical Engineering Journal 458 (2023): 141350, 10.1016/j.cej.2023.141350.

[16] N. Verdier , S. El Khakani , D. Lepage , et al., “Polyacrylonitrile‐Based Rubber (HNBR) as a New Potential Elastomeric Binder for Lithium‐Ion Battery Electrodes,” Journal of Power Sources 440 (2019): 227111, 10.1016/j.jpowsour.2019.227111.

[17] A. K. Bhowmick and S. Saha , “Smart Thermoplastic Elastomers With High Extensibility From Poly (Vinylidene Fluoride) and Hydrogenated Nitrile Rubber: Processing–Structure–Property Relationship,” Rubber Chemistry and Technology 91 (2018): 268–295, 10.5254/rct-18-82676.

[18] J. Ahn , B. Park , J. Kim , M. K. Um , J. W. Yi , and J. K. Yoo , “Multifunctional Additives for High‐Energy‐Density Lithium‐Ion Batteries: Improved Conductive Additive/Binder Networks and Enhanced Electrochemical Properties,” ACS Applied Materials & Interfaces 13 (2021): 19970–19982, 10.1021/acsami.1c00848. 33880915

[19] A. Park , S. Kim , J.‐Y. Jung , et al., “Characterization of Interactions in Battery Slurry via MD simulation: Influence on Miscibility, Morphology, and Dispersion With Varying ACN Content in HNBR,” The Journal of Chemical Physics 161 (2024): 234905, 10.1063/5.0244629. 39679525

[20] S. Saha and A. K. Bhowmick , “Effect of structure development on the rheological properties of PVDF/HNBR‐based thermoplastic elastomer and its vulcanizates,” Journal of Applied Polymer Science 137 (2019): 48758, 10.1002/app.48758.

[21] G. Zhong , L. Zhang , R. Su , K. Wang , H. Fong , and L. Zhu , “Understanding Polymorphism Formation in Electrospun Fibers of Immiscible Poly(vinylidene fluoride) Blends,” Polymer 52 (2011): 2228–2237, 10.1016/j.polymer.2011.03.024.

[22] N. D. Govinna , I. Sadeghi , C. Schick , A. Asatekin , and P. Cebe , “Crystallization Kinetics, Polymorphism Fine Tuning, and Rigid Amorphous Fraction of Poly(vinylidene fluoride) Blends,” Polymer Crystallization 4 (2021): 10205, 10.1002/pcr2.10205.

[23] S. Jang , Y. Kang , H. Kim , J. Park , and K. T. Lee , “Digital Twin Reveals the Impact of Carbon Binder Domain Distribution on Performance of Lithium‐Ion Battery Cathodes,” Small Structures 6 (2024): 2400350, 10.1002/sstr.202400350.

[24] X. Gao and J. Xu , “Carbon Binder Domain Inhomogeneity in Silicon‐Monoxide/Graphite Composite Anode by 2D Multiphysics Modeling,” Advanced Science 11 (2024): 2400729, 10.1002/advs.202400729. 38774942 PMC11304268

[25] M. M. Abolhasani , M. Ashjari , S. Azimi , and H. Fashandi , “Investigation of an Abnormal α Polymorph Formation in Miscible PVDF Nanocomposite Blend Using Kinetics of Crystallization,” Macromolecular Chemistry and Physics 217 (2015): 543–553, 10.1002/macp.201500344.

[26] B. J. Landi , M. J. Ganter , C. D. Cress , R. A. DiLeo , and R. P. Raffaelle , “Carbon Nanotubes for Lithium Ion Batteries,” Energy & Environmental Science 2 (2009): 638–654, 10.1039/b904116h.

[27] W. Sun and C. Huang , “Exploring the Role of Carbon Binder Domain Morphology in Enhancing the Electrochemical Performance of Li‐Ion Battery,” Journal of Power Sources 641 (2025): 236904, 10.1016/j.jpowsour.2025.236904.

[28] S. Parija and A. R. Bhattacharyya , “Role of Interfacial Interactions to Control the Extent of Wrapping of Polymer Chains on Multi‐Walled Carbon Nanotubes,” RSC Advances 6 (2016): 42334–42346, 10.1039/c6ra06258j.

[29] J. F. Cardenas and A. Gromov , “The effect of bundling on the G′ Raman band of single‐walled carbon nanotubes,” Nanotechnology 20 (2009): 465703, 10.1088/0957-4484/20/46/465703. 19843989

[30] J. F. Baumgärtner , K. V. Kravchyk , and M. V. Kovalenko , “Navigating the Carbon Maze: A Roadmap to Effective Carbon Conductive Networks for Lithium‐Ion Batteries,” Advanced Energy Materials 15 (2024): 2400499, 10.1002/aenm.202400499.

[31] T. Beuse , M. Fingerle , C. Wagner , M. Winter , and M. Borner , “Comprehensive Insights Into the Porosity of Lithium‐Ion Battery Electrodes: A Comparative Study on Positive Electrodes Based on LiNi0.6Mn0.2Co0.2O2 (NMC622),” Batteries 7 (2021): 70, 10.3390/batteries7040070.

[32] L. Froboese , P. Titscher , B. Westphal , W. Haselrieder , and A. Kwade , “Mercury intrusion for ion‐ and conversion‐based battery electrodes—Structure and diffusion coefficient determination,” Materials Characterization 133 (2017): 102–111, 10.1016/j.matchar.2017.09.002.

[33] Z. Yan , L. Wang , H. Zhang , and X. He , “Determination and Engineering of Li‐Ion Tortuosity in Electrode Toward High Performance of Li‐Ion Batteries,” Advanced Energy Materials 14 (2024): 2303206, 10.1002/aenm.202303206.

[34] J. Landesfeind , J. Hattendorff , A. Ehrl , W. A. Wall , and H. A. Gasteiger , “Tortuosity Determination of Battery Electrodes and Separators by Impedance Spectroscopy,” Journal of The Electrochemical Society 163 (2016): A1373–A1387, 10.1149/2.1141607jes.

[35] Z. A. Khan , M. Agnaou , M. A. Sadeghi , A. Elkamel , and J. T. Gostick , “Pore Network Modelling of Galvanostatic Discharge Behaviour of Lithium‐Ion Battery Cathodes,” Journal of The Electrochemical Society 168 (2021): 070534, 10.1149/1945-7111/ac120c.

[36] H. Luo , J. Zhu , E. Sahraei , and Y. Xia , “Adhesion Strength of the Cathode in Lithium‐Ion Batteries Under Combined Tension/Shear Loadings,” RSC Advances 8 (2018): 3996–4005, 10.1039/c7ra12382e.

[37] H. Gruhn , A. Rajic , T. Krueger , M. Mund , and M. W. Kandula , “Mechanical Testing of Battery Electrodes by Pull‐off and Peel Tests: A Comparative Study,” Journal of Advanced Joining Processes 13 (2026): 100378, 10.1016/j.jajp.2026.100378.

[38] F. Gardea , B. Glaz , J. Riddick , D. C. Lagoudas , and M. Naraghi , “Energy Dissipation due to Interfacial Slip in Nanocomposites Reinforced With Aligned Carbon Nanotubes,” ACS Applied Materials & Interfaces 7 (2015): 9725–9735, 10.1021/acsami.5b01459. 25905718

[39] I. Srivastava , D. S. Bolintineanu , J. B. Lechman , and S. A. Roberts , “Controlling Binder Adhesion to Impact Electrode Mesostructures and Transport,” ACS Applied Materials & Interfaces 12 (2020): 34919–34930, 10.1021/acsami.0c08251. 32613823

[40] X. Cai , T. Lei , D. Sun , and L. Lin , “A critical analysis of the α, β and γ phases in poly(vinylidene fluoride) using FTIR,” RSC Advances 7 (2017): 15382–15389, 10.1039/c7ra01267e.

[41] R. Gregorio , “Determination of the α, β, and γ crystalline phases of poly(vinylidene fluoride) films prepared at different conditions,” Journal of Applied Polymer Science 100 (2006): 3272–3279, 10.1002/app.23137.

[42] A. S. Jayasekara and P. Cebe , “Quantitative analysis of polar crystalline fractions in poly(vinylidene fluoride) electrospun fibers and electrosprayed films,” Polymer 281 (2023): 126140, 10.1016/j.polymer.2023.126140.

[43] C. Fu , H. Zhu , N. Hoshino , T. Akutagawa , and M. Mitsuishi , “Interfacial Nanostructuring of Poly(vinylidene fluoride) Homopolymer With Predominant Ferroelectric Phases,” Langmuir 36 (2020): 14083–14091, 10.1021/acs.langmuir.0c02667. 33147043

[44] J. Mijovic , J.‐W. Sy , and T. K. Kwei , “Reorientational Dynamics of Dipoles in Poly(vinylidene fluoride)/Poly(methyl methacrylate) (PVDF/PMMA) Blends by Dielectric Spectroscopy,” Macromolecules 30 (1997): 3042–3050, 10.1021/ma961774w.

[45] R. P. S. Chakradhar , G. Prasad , P. Bera , and C. Anandan , “Stable superhydrophobic coatings using PVDF–MWCNT nanocomposite,” Applied Surface Science 301 (2014): 208–215, 10.1016/j.apsusc.2014.02.044.

[46] O. García‐Zaldívar , T. Escamilla‐Díaz , M. Ramírez‐Cardona , et al., “Ferroelectric‐Paraelectric Transition in a Membrane With Quenched‐ Induced δ‐Phase of PVDF,” Scientific Reports 7 (2017): 5566, 10.1038/s41598-017-06044-y. 28717213 PMC5514063

[47] Z. Guo , E. Nilsson , M. Rigdahl , and B. Hagström , “Melt Spinning of PVDF Fibers With Enhanced *β* Phase Structure,” Journal of Applied Polymer Science 130 (2013): 2603–2609, 10.1002/app.39484.

[48] N. Ahbab , S. Naz , T. B. Xu , and S. Zhang , “A Comprehensive Review of Piezoelectric PVDF Polymer Fabrications and Characteristics,” Micromachines 16 (2025): 386, 10.3390/mi16040386. 40283263 PMC12029650

[49] Y. Li , G. Zhang , S. Song , H. Xu , M. Pan , and G. J. Zhong , “How Chain Intermixing Dictates the Polymorphism of PVDF in Poly(vinylidene fluoride)/Polymethylmethacrylate Binary System During Recrystallization: A Comparative Study on Core–Shell Particles and Latex Blend,” Polymers 9 (2017): 448, 10.3390/polym9090448. 30965749 PMC6418962

[50] S. Stoyanova , E. Ivanov , L. R. Hegde , et al., “PVDF Hybrid Nanocomposites With Graphene and Carbon Nanotubes and Their Thermoresistive and Joule Heating Properties,” Nanomaterials 14 (2024): 901, 10.3390/nano14110901. 38869526 PMC11173396

[51] S. Mireja and D. V. Khakhar , “Relative Thermodynamic Stability of α and *β* PVDF Crystal Phases: A Molecular Simulation Methodology,” The Journal of Physical Chemistry B 129 (2025): 6975–6984, 10.1021/acs.jpcb.5c01058. 40577598

[52] S. Mireja and D. V. Khakhar , “Molecular Simulation of the Melting of PVDF Crystal Phases,” Computational Materials Science 250 (2025): 113690, 10.1016/j.commatsci.2025.113690.

[53] H. Zhang , M. Zhao , Z.‐X. Huang , and J.‐P. Qu , “Synergistic Effect Based on Enhanced Local Shear Forces in PVDF/TiO_2_/CNT Ternary Composites,” Industrial & Engineering Chemistry Research 59 (2020): 18887–18897, 10.1021/acs.iecr.0c03725.

[54] Y. Jiang , D. Wen , Q. Xu , J. Lv , R. Zhao , and P. Peng , “The Origin of the Anomalous Expansion of the First Peak in the Radial Distribution Function During the Rapid Solidification of Tantalum Metal,” Physical Chemistry Chemical Physics 27 (2025): 13280–13294, 10.1039/D5CP00247H. 40421810

[55] J. Cui , J. Zhao , S. Wang , and Y. Li , “A Comparative Study on Enhancement of Mechanical Andtribological Properties of Nitrile Rubber Composites Reinforced by Different Functionalized Graphene Sheets:Molecular Dynamics Simulations,” Polymer Composites 42 (2020): 205–219, 10.1002/pc.25819.

[56] M. R. Milovanovic , I. M. Stankovic , J. M. Zivkovic , D. B. Ninkovic , M. B. Hall , and S. D. Zaric , “Water: New Aspect of Hydrogen Bonding in the Solid State,” IUCrJ 9 (2020): 639–647, 10.1107/S2052252522006728. PMC9438494 36071797

[57] T. K. Patra and J. K. Singh , “Coarse‐Grain Molecular Dynamics Simulations of Nanoparticle‐Polymer Melt: Dispersion vs. Agglomeration,” The Journal of Chemical Physics 138 (2013): 144901, 10.1063/1.4799265. 24981543

[58] S. Kim , H. Lee , J. Lim , J. Park , and Y. M. Lee , “Digital Twin Battery Modeling and Simulations: A New Analysis and Design Tool for Rechargeable Batteries,” ACS Energy Letters 9 (2024): 5225–5239, 10.1021/acsenergylett.4c01931.

[59] J. Lim , J. Choi , K. G. Kim , J. Song , H. Lee , and Y. M. Lee , “Digital Twin‐Driven Mechanical Degradation Diagnostics: Unraveling Microstructure Evolution of Silicon‐based Lithium‐Ion Battery Anodes,” Small 22 (2026): 07883, 10.1002/smll.202507883. 41288140

[60] J. Song , R. C. Ihuaenyi , J. Lim , et al., “A microstructural electrochemo‐mechanical model of high‐nickel composite electrodes towards digital twins to bridge the particle and electrode‐level characterizations,” Energy & Environmental Science 18 (2025): 3129–3147, 10.1039/d4ee04856c.

[61] J.‐K. Yoo , Y. Oh , T. Park , K. E. Lee , M.‐K. Um , and J.‐W. Yi , “Optimization of Carbon Nanotubes as Conductive Additives for High‐Energy‐Density Electrodes for Lithium‐Ion Batteries,” Energy Technology 7 (2019): 1800845, 10.1002/ente.201800845.

[62] B. J. Jeon , H. Jeong , S. Yoon , S. Park , J. Im , and K. M. Jeong , “Thick Electrode Design Enabled by a Carbon–Binder Domain–Resolved Dual‐Pore Transmission Line Model for Lithium‐Ion Batteries,” Advanced Energy Materials 16 (2025): 05334, 10.1002/aenm.202505334.

[63] J. K. Park , W. Shin , W. Jo , et al., “Ultrahigh‐Mass‐Loading Electrodes With Enhanced Homogeneity Using a High‐Concentration Slurry for Lithium‐Ion Batteries,” Carbon Energy 8 (2026): c70108, 10.1002/cey2.70108.

[64] J. Ali , T. J. Embleton , J. H. Choi , et al., “Overcoming Through‐Plane Resistance in Lithium‐Ion Battery Cathode Electrodes via the Application of Trace High‐Aspect‐Ratio Carbon Nanofiber Carbon Additives With Carbon Nanotube‐Coated LiNi 0.8 Co 0.1 Mn 0.1 O 2,” ACS Applied Energy Materials 7 (2024): 10134–10148, 10.1021/acsaem.4c02269.

[65] P. Gasper , N. Sunderlin , N. Dunlap , et al., “Lithium Loss, Resistance Growth, Electrode Expansion, Gas Evolution, and Li Plating: Analyzing Performance and Failure of Commercial Large‐Format NMC‐Gr Lithium‐Ion Pouch Cells,” Journal of Power Sources 604 (2024): 234494, 10.1016/j.jpowsour.2024.234494.

[66] Y. Chen , J. Key , K. O'Regan , T. Song , Y. Han , and E. Kendrick , “Revealing the Rate‐Limiting Electrode of lithium batteries at high rates and mass loadings,” Chemical Engineering Journal 450 (2022): 138275, 10.1016/j.cej.2022.138275.

[67] H. Xiang , H. Ren , Y. Liu , D. Yang , and X. Feng , “High Loading Electrode With Superior Electron/Ion Transport Network for High Performance Lithium‐Ion Batteries,” Journal of Power Sources 609 (2024): 234672, 10.1016/j.jpowsour.2024.234672.

[68] J. Wu , X. Zhang , Z. Ju , et al., “From Fundamental Understanding to Engineering Design of High‐Performance Thick Electrodes for Scalable Energy‐Storage Systems,” Advanced Materials 33 (2021): 2101275, 10.1002/adma.202101275. 34028911

[69] M. G. Nam , S. W. Jeong , and P. J. Yoo , “Advancing Post‐Secondary Batteries Under Lean Electrolyte Conditions Through Interfacial Modification Strategies,” Advanced Energy Materials 15 (2025): 2400035, 10.1002/aenm.202400035.

[70] E. N. Primo , M. Chouchane , M. Touzin , P. Vazquez , and A. A. Franco , “Understanding the calendering processability of Li(Ni_0.33_Mn_0.33_Co_0.33_) O_2_‐based cathodes,” Journal of Power Sources 488 (2021): 229361, 10.1016/j.jpowsour.2020.229361.

[71] M. Abdollahifar , H. Cavers , S. Scheffler , A. Diener , M. Lippke , and A. Kwade , “Insights Into Influencing Electrode Calendering on the Battery Performance,” Advanced Energy Materials 13 (2023): 2300973, 10.1002/aenm.202300973.

