# Supplementary material for: A Dispersant‐Driven Carbon‐Binder Domains Homogeneity in High‐Energy‐Density Electrodes for Lithium‐Ion Batteries
Source: Adv Sci (Weinh). 2026 Aug 18:e77293. Online ahead of print. doi: 10.1002/advs.77293 (PMC13483137; doi:10.1002/advs.77293)
Supplement: Supplementary file 1 [file ADVS-9999-e77293-s001.docx]

Supporting Information

A Dispersant-Driven Carbon-Binder Domains Homogeneity in High-Energy-Density Electrodes for Lithium-Ion Batteries

Da-Sol Kwon^1,2†^, Min Young Seo^3†^, Jinho Ahn^4 †^, Yea-Ji Jeong^5^, Woohyeon Shin^1^, Wonseok Ko^4^, Min Jeong Kim^1,6^, Jiny Lee^1,4^, Won-Yong Jeon^5^, Min-Jae Choi^7^, Kwang In Kim^8^, Jin Ju Eom^8^, Sang Yeop Oh^8^, Honghee Lee^9^, Joona Bang^2^, Jongsoon Kim^4,10*^, YongJoo Kim^3*^, and Jung-Keun Yoo^1*^

D.-S. Kwon, W. Shin, M. J. Kim, J. Lee, J. -K. Yoo*

^1^ Energy Storage Research Center, Korea Institute of Science and Technology (KIST), 14 Gil 5 Hwarang-ro, Seongbuk-gu, Seoul 02792, Republic of Korea

E-mail: yoojk@kist.re.kr (J.-K. Yoo)

D.-S. Kwon, J. Bang

^2^ Department of Chemical and Biological Engineering, Korea University, 145, Anam-ro, Seongbuk-gu, Seoul 02841, Republic of Korea

M. Y. Seo, Y. Kim*

^3^ Department of Materials Science and Engineering, Korea University, 145, Anam-ro, Seongbuk-gu, Seoul 02841, Republic of Korea

E-mail: cjyjee@korea.ac.kr (Y. Kim)

J. Ahn, W. Ko, J. Lee, J. Kim*

^4^ Department of Energy Science, Sungkyunkwan University, 2066, Seobu-ro, Jangan-gu, Suwon, Gyeonggi-do, 16419, Republic of Korea

E-mail: jongsoonkim@skku.edu (J. Kim)

Y.-J. Jeong, W.-Y. Jeon

^5^ Department of Chemical and Biochemical Engineering, Dongguk University, 30, Pildong-ro-1-gil, Jung-gu, Seoul, 04620, Republic of Korea

M. J. Kim

^6^ Department of Energy Engineering, Hanyang University, 222, Wangsimni-ro, Seongdong-gu, Seoul 04763, Republic of Korea

M.-J. Choi

^7^ Department of Advanced Materials Science and Engineering, Sungkyunkwan University, 2066, Seobu-ro, Jangan-gu, Suwon, Gyeonggi-do, 16419, Republic of Korea

K. I. Kim, J. J. Eom, S. Y. Oh

^8^ Battery Materials R&D Center, Hansol Chemical, 126-25 Sandanoryoung-gil, Samgi-myeon, Iksan-si, Jeonbuk-do 54524, Republic of Korea

H. Lee

^9^ Multiphysics Digital Twin Lab, TRINITY Engineering Co., Ltd., Busan 48059, Republic of Korea

J. Kim*

^10^ SKKU Institute of Energy Science and Technology (SIEST), Sungkyunkwan University, 2066, Seobu-ro, Jangan-gu, Suwon, Gyeonggi-do, 16419, Republic of Korea
E-mail: jongsoonkim@skku.edu (J. Kim)

*Characterization of Electronic and Ionic Transport in CBD:* C-AFM was performed using an XE-100 (Park System, Korea) equipped with a CDT-CONTR conductive cantilever to visualize the electronic conductive distribution on the electrode surface. The distribution of CBD across the electrode cross-section was analyzed using EPMA (JXA-8500F, JEOL Ltd., Japan) in qualitative mapping mode at 15 kV accelerating voltage. EPMA analysis was carried out on a cross-sectional electrode prepared by ion beam milling. Raman spectroscopy (LabRAM HR Evolution, HORIBA, Japan) was conducted using a 532 and 785 nm excitation laser to analyze the structural features. Optical microscopy (HNM001, HiMax Tech, Korea) was employed to investigate the dispersion state in CNT–PVDF–dispersant composite films. These films were fabricated by casting a solution with the same composition as the slurry, excluding the active material, and dried under the same conditions used for electrode preparation. Electrode resistance was measured using a Hioki electrode resistance meter (XF-057, Hioki, Japan) under a constant current of 10 mA. The Al foil resistance (2.65 ×10^–6^ Ω cm^–1^) and the active layer thickness (approximately 35 *μ*m) were set as reference parameters to calculate the active layer and interfacial resistance. The two-dimensional microstructures of the electrodes were observed using SEM (Regulus 8230, Hitachi, Japan). The multiple SEM images were analyzed using ImageJ to quantitatively evaluate the microstructure. For three-dimensional analysis of pore structure within the electrode, X-ray CT (CT-ALPHA nanotube, ProCon X-Ray GmbH, Germany) was performed with a spatial resolution of approximately 150 nm. The X-ray CT data were processed and analyzed using VGSudio Max, and the cross-sectional slices of the 3D reconstructed CT data were extracted and visualized using MyVGL for detailed examination of the internal pore structure. MacMullin number and tortuosity were calculated from EIS analysis of the cathode symmetric cell, which was conducted in a frequency range of 1 MHz to 100 mHz with an amplitude of 5 mV. Further details are provided in the Supporting Information. For Ionic conductivity, the freestanding electrode was sandwiched between two separators and Li metal electrodes in an electron-blocking configuration, and 200 *μ*L electrolyte was added to the cell. EIS was performed from 1 MHz to 100 mHz with an amplitude of 10 mV to obtain the ionic resistance. For electronic conductivity, the freestanding electrode was assembled in a stainless-steel/electrode/stainless-steel configuration, and the current response was recorded for 1 h under DC voltage sweep from -50 to 50 mV. The electronic conductivity was calculated from the resulting current-voltage response. To characterize the pore structure of the composite electrodes, the Brunauer-Emmett-Teller (BET) specific surface area was analyzed using an ASAP 2020 Instrument (Micromeritics, USA) from nitrogen adsorption-desorption isotherms collected at 77 K. The larger-scale pore structure was further examined by mercury intrusion porosimetry using an AutoPore IV system (Micrometrics, USA).

*Mechanical and Structural Characterizations:* The 90° adhesion tests were conducted using the Lloyd-LS1 universal tensile machine (Lloyd, UK) with a crosshead speed of 100 mm min^–1^ of the electrode was measured using the digital material tester (Lloyd-LS1). The 3 M double-sided tape was attached to the electrode with a width of 20 mm using a rubber roller. The tensile tests were performed using Lloyd-LS1 to assess the mechanical strength of PVDF–dispersant and CBD composite films with a cross-head speed of 10 mm min^–1^. The composition and preparation conditions of these films were identical to those of the electrode. The samples were shaped into dog-bone-shaped specimens in accordance with the ASTM standard D638-M (Type V specimens). The adhesion test and tensile test were conducted more than three times to ensure consistency of the results. Rheological measurements were conducted using a Discovery Hybrid Rheomter (DHR-2, TA Instruments, USA) with a 8 mm parallel plate geometry with a loading gap of 300 μm at 23 ℃. Shear-rate-dependent viscosity was evaluated via flow sweep in the shear rate range of 10^–2^ to 10^–3^ s^–1^, while viscoelastic properties were analyzed through frequency sweep tests conducted from 10^–1^ to 10^2^ rad s^–1^ at a constant strain of 0.1%. These measurements were applied to slurry, CNT dispersion, PVDF–dispersant, and CNT–PVDF–dispersant solution at the same concentration. The FTIR spectra of the films were obtained by Nicolet iS10 (Thermo Scientific, USA) with a range of 700 to 2000 cm^–1^. The crystalline phase of PVDF and the changes in PVDF crystallinity, influenced by the dispersant and the presence or absence of CNT, were confirmed. The XRD was carried out on a X’Per3-Powder (Malvern PANalytical, Netherlands) with Cu–K*α* radiation (λ=1.54060 Å), at a scanning rate of 2.5° min^–1^ and step size 0.02 over the range of 2θ = 15-45° at room temperature. The DSC analysis was conducted using a DSC25 (TA Instruments, USA) with a heating rate of 10 ℃ min^–1^ from 25 to 180 ℃. The crystallinity behavior was analyzed by examining the second cycle to eliminate thermal history effects. X-ray photoelectron spectroscopy (XPS) was performed using a Nexsa (Thermo Fisher Scientific Inc., USA) with a microfocus monochromatic Al Kα X-ray source. The electrode surface was sputtered once with Ar^+^ ions to remove surface contaminants.

*Molecular Dynamics (MD) Simulations:* All atom MD simulations were performed using the GROMACS molecular dynamics simulation package to investigate how dispersants influence the molecular configuration of PVDF and their intermolecular interactions induced by different dispersants. In this study, two types of systems were modeled: PVDF–dispersant (either HNBR or AFPA) and CNT–PVDF–dispersant. The simulation systems were constructed to reflect the electrode composition ratio employed in the experiments. The compositions were set to CNT:PVDF:dispersant = 0.425:1.5:0.21 wt% for the CNT-containing system, and PVDF:dispersant = 1.5:0.21 wt% for the CNT-free system, respectively.

The following molecular structures were used for PVDF, HNBR, AFPA, and CNT in MD simulation. The PVDF (C_30_H_31_F_31_) structure was modeled with 15 repeating units of (CH_2_CF_2_), with terminal hydrogen and fluorine atoms added to complete the valence of the terminal atoms. HNBR (C_27_H_47_N_3_) consists of 7 monomer units, comprising 3 acrylonitrile and 4 butadiene. Among the butadiene units, three were fully hydrogenated to (CH_2_CH_2_) segments, while the remaining unit retained its unsaturated structure to match the overall elemental composition. AFPA (C_66_H_78_O_12_) included 12 monomers equally divided between 6 polyacrylate and 6 polystyrene units. The structures of CNTs were constructed using a (4,4) chiral index with a length of 3nm. The molecular formulas and images of the molecules are provided in **Figure S29**.

To reflect the experimental formulation, all simulation systems were constructed by adjusting the number of molecules while maintaining a total mass density of 1.0 g cm^-3^. In addition to the experimental compositions, we ran additional simulations with different numbers of dispersants to understand the overall phase behavior of the system. For the PVDF–dispersant system, simulations were performed in a stepwise manner by gradually increasing the number of dispersant molecules relative to PVDF. In the PVDF–HNBR system, the number of HNBR was increased from 0 to 39, whereas in the PVDF–AFPA system, the number of AFPA molecules ranged from 0 to 15. For CNT–PVDF–dispersant systems, a fixed composition was directly adopted from the electrode composition, and a total of 11 CNTs were included are randomly distributed within the simulation box.

With the system compositions defined, MD simulations were carried out under the conditions described below. Force field parameters for PVDF, HNBR, and AFPA were generated by LigParGen force field generator based on optimized potentials for liquid simulation-all-atom (OPLS-AA) force field. The initial configurations of all systems were generated using PACMOL. For all systems, after energy minimization, 10 ns NPT run at 353 K was performed for equilibration followed by a short 1 ns NVT run at 353 K to escape from any possible metastable states. A final 30 ns NVT production run at 353 K was carried out and the last 2 ns trajectory was used in phase analysis and RDF analysis. The cutoff distance for short-range coulomb and van der Waals interactions was set to 1 nm. The particle mesh Ewald method was used for long-range interactions further away than the cutoff distance 1.0 nm, which was also applied to both electrostatic and van der Waals interactions. A fourth-order (cubic) interpolation scheme was used to assign charges to the mesh, and the Fourier grid spacing was set to 0.16 nm for accurate reciprocal-space calculations. LINear Constraint Solver (LINCS) algorithm was used for constraining bond lengths and the integration time step of 2 fs was used for both Nosé–Hoover thermostat and Parrinello–Rahman pressure coupling during the simulation.

*Digital twin analysis:* All simulations for properties related to the electrode were performed using Geodict software. 3D electrode models were reconstructed by Geodict software based on hundreds of electrode side images obtained by FIB-SEM analysis. To systematically analyze the electrode microstructure of the 3D electrode model, various Geodict functions were used to image the CBD distribution, pores within the geometric structure, lithium-ion diffusion channels, and electron transport channels. In addition, porosity, lithium-ion diffusivity, lithium-ion diffusion tortuosity, electrical conductivity, and electrical resistance were simulated to derive quantitative values.

**Figure S1.** Photographs shwoing time-dependent phase-separation in PVDF–dispersant blends, used to assess PVDF–dispersant compatibility under quiescent conditions. (a) PVDF–HNBR solution in NMP. (b) PVDF–AFPA solution in NMP. Images were acquired at 0 h and after 24 hours at rest.

**Figure S2.** Rheological properties of CNT–dispersant dispersions and slurries prepared using HNBR and AFPA as dispersants, respectively. (a) Flow behaviors of CNT dispersions and slurries. (b) Corresponding yield stress estimated from (a) using the Casson model. (c) Complex modulus and phase angle at angular frequency 6.3 rad s^–1^ for CNT dispersions and slurries. All rheological measurements were performed at 23 ℃.

Rheological measurements were conducted for both CNT dispersants and slurries containing either HNBR and AFPA dispersants. As shown in **Figure S2a**, all samples exhibited shear-thinning behavior, and among them, the AFPA-based samples showed a steeper slope, indicating a more uniform dispersion and a microstructure with higher sensitivity to shear deformation.^[1]^ Yield stress were obtained from flow behavior using the Casson model, serving as a measure of dispersion stability (**Figure S2b**). While the AFPA-based slurry exhibited a lower yield stress, indicating better dispersion, its corresponding CNT dispersion showed higher yield stress, which may be attributed to stronger interactions and amore rigid network structure, which reduced flowability.^[2-3]^ The strong interaction between CNT and AFPA is further supported by the phase angle results in **Figure S2c**. While all samples exhibit solid-like behavior (*G’ > G"*), the CNT–AFPA dispersion presents the lowest phase angle, indicating the most pronounced elastic character. Since lower phase angles correspond to increased elasticity, this suggests that the CNT–AFPA dispersion forms a more rigid and interconnected network.^[4]^

**Figure S3.** SEM images revealing the local distribution of CNT and PVDF on the surface and between NCM particles in electrodes prepared with (a) HNBR (b) AFPA dispersants.

**Figure S4.** Characterization of CNT dispersion quality and local CNT environment in CBD films and electrodes. (a, b) Optical microscope (OM) images of the CBD films based on HNBR and AFPA, with inset photographs showing macroscopic dispersion. Raman spectra of (c) CBD films (d) practical composite electrodes prepared with HNBR and AFPA.

Raman spectroscopy was used to compare the local environment in CBD films and practical composite electrodes (**Figure S4**). Although I_D_/I_G_ ratio is commonly used as a defect-related indicator for CNTs, Raman spectra of CNTs can also be affected by bundling and the surrounding matrix.^[5]^ In the CBD films, the AFPA-based sample showed a more pronounced G-band blu shift and increased the I_D_/I_G_ ratio, supporting stronger CNT-PVDF interaction and improved CNT de-bundling.^[6]^ When examined in the AFPA- and HNBR-based electrodes, the I_D_/I_G_ ratio were comparable, indicating that the defect-related CNT structure was not significantly altered at the electrode level. Meanwhile, the AFPA-based electrode exhibited a more define G‘/2D-band feature, and this narrower band supports reduced heterogeneity in the local CNT environment.^[7]^

**Figure S5.** Comparison of electronic resistance in electrodes with HNBR and AFPA dispersants. (a) Bulk resistivity of the active material layer, (b) Interfacial resistance between the electrode and the current collector. All data are averaged over three repeated measurements to ensure reproducibility.

**Figure S6.** XPS analysis of the NCM-associated CBD-related species near NCM particles in electrodes. (a) Atomic ratios of C 1*s*, F 1*s*, O 1*s*, and Ni 2*p* for pristine and surface-etched HNBR- and AFPA-based electrodes. (b) O 1*s* (c) C 1*s* spectra of the etched electrodes.

XPS analysis was performed before and after mild surface etching to examine the CBD composition near NCM particles. The atomic ratio analysis showed a consistent dispersant-dependent difference before and after etching. The increased Ni 2*p* contribution after etching indicates that the etched region became closer to the NCM surface (**Figure S6a**). In both pristine and etched states, the AFPA-based electrode exhibited a higher C fraction, whereas the HNBR-based electrode showed a larger F contribution.

The O 1s spectra further confirmed the exposure of the NCM-adjacent region after etching, as evidenced by the increased NCM lattice oxygen contribution and reduced organic oxygen species (**Figure S6b**).^[8]^ Notably, although the HNBR-based electrode exhibited a lower overall C fraction, the CF_2_ bond at 290 eV, indicating a stronger PVDF-derived binder character near NCM (**Figure S6c**).^[9]^ Together, these results suggest that the AFPA-based electrode forms a more carbonaceous CBD environment around NCM particles, whereas the HNBR-based electrode is more enriched with PVDF-derived fluorinated species near NCM.

**Figure S7.** Shear-dependent rheological behavior of PVDF–dispersants and CNT–PVDF–dispersants solutions prepared with either HNBR or AFPA at the same concentration. (a) Viscosity as a function of shear rate. (b) Yield stress values before and after CNT introduction. The rheological tests were carried out at a controlled temperature of 23 ℃.

The AFPA-based samples showed a greater reduction in yield stress after CNT addition than the HNBR-based samples in the CBD solution containing PVDF (**Figure S7b**). This suggests that CNTs were more uniformly embedded within the polymer matrix in the AFPA system, resulting in a more flowable structure.^[10-11]^ In contrast, CNT dispersion without binder showed increased yield stress with better dispersion, as CNT networks dominated the flow resistance. These results indicate that while the rheology of binder-free systems in primarily governed by CNT networks, the presence of PVDF shifts the dominant factor to the binder matrix.

**Figure S8.** Cross-sectional SEM images and corresponding pore size distributions of electrodes based on HNBR (a, c) and AFPA (b, d) dispersants. In SEM images, black color regions represent pores between primary NCM particles. Pore size analysis was obtained through Image J software from three representative SEM images per sample. The inset in the distribution plots were added to highlight differences in pore counts at larger sizes.

**Figure S9.** Pore-structure characterization of HNBR- and AFPA-electrodes. (a) N_2_ adsorption isotherms. (b) Specific pore volume and pore porosity determined by MIP. (c) Mercury intrusion pore-size distributions.

**Figure S10.** (a) Nyquist plots from symmetric-cell EIS of electrodes prepared with HNBR and AFPA dispersants. (b) Ionic resistance extracted from the EIS data. (c) MacMullin number calculated from the effective ionic conductivity derived from (b). Detailed methods and calculation procedures are described in the supporting information.

To suppress the influence of charge-transfer resistance and focus on ionic transport within the electrode structure, symmetric cells were assembled using 50 mm tetrabutylammonium perchloate (TBAClO_4_) in EC/DMC (1:1 w/w) as the electrolyte.^[12-13]^ EIS revealed a clear difference in ion-transport-related resistance between HNBR and AFPA-based electrodes. Based on the transmission-line model, the ionic resistance (*R*_ion_) was calculated using the equation:

*R*_ion_ = 3 × (*R*_low_ – *R*_HFR_)

where *R*_low_ is the low-frequency intercept and *R*_HFR_ is the high-frequency resistance.^[12]^ The effective ionic conductivity (*σ*_eff_) within the electrode was then determined by incorporating the measured ionic resistance, electrode thickness (*L*), and area (*A*), according to:

*σ*_eff_ = *L* / (*R*_ion_ × *A*)

Finally, the MacMullin number was calculated as the ratio of the ionic conductivity of the bulk electrolyte (*σ*_bulk_ = 1.74 mS cm^–1^) to the effective ionic conductivity (*σ*_eff_) measured within the electrode.^[12]^

*N*_m_ = *σ*_bulk_ / *σ*_eff_

**A lower** *N*_m_ **indicates reduced ion conduction resistance and improved pore connectivity.**

**Figure S11.** (a) Nyquist plots from symmetric-cell EIS of NCM electrodes prepared with HNBR and AFPA dispersants, measured using 1  m LiPF₆ in EC/DMC (1:1 v/v) as the electrolyte. The high-frequency region is enlarged in the inset.

**The enhanced pore connectivity facilitates ion transport by ensuring uniform electrolyte filling within the porous network, which reduces local resistance variations and improves charge transfer kinetics at the electrode/electrolyte interface.^[14-15]^ EIS of the symmetric cell confirmed that the** AFPA-based electrode **exhibited significantly lower charge transfer resistance and a steeper Warburg impedance slope than HNBR**-based electrode**, indicating more efficient interfacial charge transfer and ion mobility.^[16-17]^**

**Figure S12.** Post-peel morphology of electrodes using HNBR (a, c, e) and AFPA (b, d, f) dispersants. (a, b) Surface photographs of electrodes after peel test. (c,d) Corresponding EDS mapping results of the tip surface, showing the distribution of Ni (green) and Al (red). (e, f) Cross-sectional SEM images highlight the detachment behavior.

**Figure S13.** Adhesion and cohesion strengths of the HNBR- and AFPA-based high loading electrodes.

**Figure S14.** A Frequency sweep results of PVDF–dispersant and CBD solution containing HNBR and AFPA dispersants. (a) Storage modulus (*G*') and loss modulus (*G*") as a function of angular frequency. (b) Complex modulus (*G**) versus phase angle (δ) at 6.3 rad s^–1^, providing a comparative measure of solid- and liquid-like characteristics depending on the dispersants and the presence of CNT.

The CNT–PVDF–AFPA solution exhibited the lowest phase angle relative to its HNBR-based counterpart, while maintaining *G*’ greater than *G*” across the entire frequency range (**Figure S14a–b**). This solid-like viscoelastic behavior indicates stronger internal cohesion and a more stable CBD network.^[18]^ The higher phase angle observed in the HNBR-based sample may result from weaker interactions between CNTs and the polymer matrix, which limit network formation and reduce elastic response.

**Figure S15.** Fluorine/NCM ratio from EPMA analysis comparing inner and outer regions of electrodes. Data represent average value from three representative spots per region.

HNBR-based electrodes exhibited a 25% increase in fluorine concentration from the inner to the outer layer, indicating binder migration to the surface. In contrast, AFPA-based electrodes maintained a uniform fluorine content of 0.09 across both layers, indicating a more homogeneous binder distribution and nearly twice the fluorine content compared with HNBR-based electrodes. The higher fluorine retention suggests a more integrated and cohesive CBD structure in the AFPA-based electrode, which may be associated with stronger PVDF–dispersant interaction that suppress binder migration.^[19]^

**Figure S16.** FT-IR based estimation of the relative *β*-phase fraction in PVDF-based composite films. (a) Magnified FT-IR spectra in 700-950cm^–1^ region, highlight the α- and *β-*phase bands at 762 cm^–1^ and 839 cm^–1^, respectively. (b) Relative *β*-phase fraction, F(*β*), calculated from the peak intensities in (a) using the equation shown in the inset.

**Figure S17.** DSC curves of PVDF–dispersant and CBD films, illustrating (a) crystallization and (b) melting temperature.

**Figure S18.** Structural characterization of pristine PVDF and PVDF–dispersant films using (a) FT-IR spectra (b) XRD pattern. A magnified view of the XRD pattern in the 14–30° region is shown to highlight differences in the PVDF phase signals.

**Figure S19.** Phase contents of PVDF calculated from MD simulations as a function of the number of dispersant molecules. (a) HNBR system, (b) AFPA system.

**Figure S20.** RDF analysis of atomic interactions with PVDF and dispersant functional groups in the (a) PVDF–HNBR and (b) PVDF–AFPA systems. Integration of RDF curves was calculated over the range of 1–5 Å, encompassing hydrogen bonding and van der Waals interaction. (c) Total integration values before and after CNT introduction.

AFPA-based CBD exhibits minimal changes upon CNT addition, with only a 0.2 % decrease in total RDF integral, indicating that the hydrogen bonded network remains largely intact. In contrast, HNBR-based CBD show a 0.795% increase, reflecting significant reorganization driven by stronger CNT–dispersant interactions.

**Figure S21.** Distribution of dispersant molecules within 5 Å of the CNT surface in CBD systems, as obtained from MD simulation. Inset snapshots illustrate representative configurations highlighting spatial interactions between CNTs and dispersants in HNBR and AFPA systems.

AFPA remains primarily associated with PVDF, with only 2 out of 15 AFPA molecules located near CNT, while PVDF remains dominant. HNBR exhibits a higher dispersant concentration around CNT, while fewer PVDF molecules remain near CNT, suggesting a transition toward CNT-driven network reorganization.

**Figure S22.** Visulization of pore-network tortuosity computed using PoroDict for electrodes prepared with HNBR and AFPA dispersants. Red stremlines indicate representative through-plane transport pathways.

**Figure S23.** Electronic conductivity, Ionic conductivity, and electrolyte uptake of HNBR- and AFPA-based cathode electrodes. (a) Direct current (DC) polarization using an ion-blocking SS/cathode/SS configuration and (b) corresponding electronic conductivities calculated from (a). (c) Nyquist plots of electrolyte-filled cathodes with TLM-based fitting and (d) corresponding ionic conductivity calculated from (c), along with electrolyte uptake after 24 h electrolyte immersion. All measurements were conducted at R.T. using 1  m LiPF_6_ in EC/DMC (1:1 v/v).

To quantitatively compare electrode-level charge transport, electronic conductivity, ionic conductivity, and electrolyte uptake were evaluated for the HNBR- and AFPA-based electrodes (**Figure S23**). Electronic resistance (*R*_e_) was obtained from the inverse slope of the linear I–V response measured using an ion-blocking SS/cathode/SS configuration, based on Ohm’s law (*V=IR*). Ionic resistance (*R*_ion_) was extracted from TLM-based fitting of the EIS spectra measured using electrolyte-filled cathodes in a Li/separator–electrolyte/cathode/separator–electrolyte/Li configuration. The corresponding conductivities were calculated using *σ*_i_ *= L/(R*_i_*A)*, where *L, R*_i_*, A* are the electrode thickness, corresponding resistance, and geometric electrode area, respectively.^[20-22]^ Electrolyte uptake was calcluated from the electrode mass increase after 24 h electrolyte immersion.

The AFPA-based electrode showed a steeper I–V slope and a smaller fitted ionic resistance than the HNBR-based electrode, resulting in higher electronic/ionic conductivity. The higher electrolyte uptake of the AFPA-based electrode suggests improved electrolyte-accessible pore connectivity, rather than simply higher total porosity, which can reduce effective ion transport tortuosity and facilitate ion transport within the cathode. These results support that AFPA-driven CBD homogenization improves both electronic and ionic transport pathways.

**Figure S24.** Electrochemical responses of CNT films prepared using as-received and purified CNTs. (a) Cyclic voltammetry (CV) profiles recorded over 3.0-4.5 V at scan rate of 0.1 mV s^–1^. (b) Linear sweep voltammetry (LSV) profiles obtained from 3.0 to 5.0 V at scan rate of 1 mV s^–1^.

**Figure S25.** Electrochemical performance of electrode containing 0.425 and 0.85 wt% CNT with HNBR and AFPA dispersants. (a) Rate capability tested at various c-rates from 0.1 C to 5 C. (b) Long-term cycling performance and coulombic efficiency under 1 C. All measurements were conducted at R.T. using 1  m LiPF₆ in EC/DMC (1:1 v/v).

**Figure S26.** EIS analysis of HNBR- and AFPA-based NCM811 electrodes before and after cycling. (a) Nyquist plots of HNBR- and AFPA-based electrodes before and after cycling. (b) Enlarged Nyquist plot after cycling.

In the Nyquist plots of NCM811‖Li half-cells, the high-frequency semicircle is commonly associated with interfacial film resistance, whereas the following middle-frequency semicircle is generally attributed to the apparent charge-transfer process coupled with double-layer capacitance (**Figure S26b**).^[22]^

**Figure S27.** High-loading NCM811 SiC full-cell performance at controlled electrolyte-to capacity (E/C) ratios. (a) Cycling performance and coulombic efficiency of HNBR- and AFPA-based full cells at an E/C ratio of 3.0 g Ah^–1^. (b) Cycling performance and coulombic efficiency at a reduced E/C ratio of 2.5 g Ah^–1^. The calculated cell-level gravimetric energy densities are indicated in the insets. (c) Corresponding voltage profiles of the full cells in (b) at selected cycles. Pouch cell with a mass loading of 30 mg cm^–2^ were cycled at a charge/discharge C-rate of 0.33 C/0.33 C at a voltage range of 2.5–4.2 V.

**Figure S28.** Rate capability and spring-back behavior of maximally calendered HNBR- and AFPA-based electrodes. (a) Rate performance of the HNBR- and AFPA-based electrodes calendered to their respective maximum electrode densities of 3.5 and 3.6 g cc^-1^. The capacities are normalized to the discharge capacity at 0.1 C. (b) Electrode thicknesses measured immediately after calendering and after one week of storage, showing the corresponding spring-back behavior.

The springback behabior of the maximally calendered electrodes was evaluated by monitoring the electrode thickness immediately after calendering and after one week of storage. The HNBR-based electrode exhibited a thickness increase from 69 to 72 *μ*m, corresponding to a springback of approximately 4.3%. In contrast, the AFPA-based electrode showed only amarginal increase from 66 to 67 *μ*m, corresponding to less than 1.5% springback. The lower degree of springback indicates that the AFPA-based electrode more effectively retained the thickness reduction achieved through calendering.

**Figure S29.** Molecular formula and their corresponding molecule images used in MD simulation.

**Table S1.** Quantitative pore shape analysis from cross-sectional SEM images of electrodes shown in Figure S6.

|  | **Feret's diameter** | **Aspect ratio** | **Perimeter** | **Circularity** |
| --- | --- | --- | --- | --- |
| HNBR | 2.14 | 2.74 | 6.29 | 0.37 |
| AFPA | 2.02 | 3.21 | 5.66 | 0.34 |

**Table S2.** Calcultaed cell-level gravimetric energy densities of the pouch full cells in Figure 7h and S26, based on all internal cell components excluding the pouch packaging film.

| **Loading**  **[mg cm^-2^]** | **Areal Capacity**  **[mAh cm^-2^]** | **Cell**  **capacity**  **[mAh]** | **Cathode**  **[mg]** | **Anode**  **[mg]** | **Separator**  **[mg]** | **Electrolyte**  **[mg]** | **Total**  **[mg]** | **Gravimetric energy density**  **[Wh kg^-1^]** |
| --- | --- | --- | --- | --- | --- | --- | --- | --- |
| 20 | 4.0 | 76.3 | 448.4 | 533.7 | 44.0 | 267.1  (E/C=3.5)^*^ | 1293.1 | 212.4 |
| 30 | 6.2 | 117.4 | 648.3 | 630.4 | 44.0 | 352.2  (E/C=3.0)^*^ | 1674.9 | 252.3 |
| 30 | 6.2 | 117.4 | 648.3 | 630.4 | 44.0 | 293.5  (E/C=2.5)^*^ | 1616.2 | 261.5 |

*Note: E/C ratio are expressed in g Ah^-1^.

**Table S3.** Calcultaed cell-level volumetric energy densities of the pouch full cells in Figure 7h and S26, based on the total internal cell thickness, including both electrodes, current collectors, and separator, while excluding the pouch packaging.

| **Loading**  **[mg cm^-2^]** | **Areal capacity**  **[mAh cm^-2^]** | **Cathode**  **[μm]** | **Anode**  **[μm]** | **Separator**  **[μm]** | **Total***  **[μm]** | **Volumetric capacity**  **[mAh cm^-3^]** | **Volumetric energy density**  **[Wh L^-1^]** |
| --- | --- | --- | --- | --- | --- | --- | --- |
| 20 | 4.0 | 77.5 | 105 | 25 | 207.5 | 192.8 | 693.9 |
| 30 | 6.0 | 112 | 112 | 25 | 249 | 240.9 | 867.5 |

*Note: The electrolyte was assumed to be fully accommodated within the electrode and separator pores and was not added separately to the internal cell volume.

**Table S4.** Extracted and transformed (pre-normalization) metrics used to construct the radar plot in Figure 7i.

|  | **1/*R*ct^a)^ @SOC0%** | **1/DCIR ^a)^ @SOC50%** | **1/Tortuosity ^a)^** | **Rate capability ^a)^**  **(5C/0.1C)** | **Cycling retention ^a)^**  **(200 cycles @1C)** |
| --- | --- | --- | --- | --- | --- |
| HNBR | 1/22 | 1/13.2 | 1/7.3 | 1/52.4 | 1/46.9 |
| AFPA | 1/4.5 | 1/10.5 | 1/2.8 | 1/76.9 | 1/67.3 |
| Source figure | Fig S9 | Fig 7g | Fig 3d | Fig S19 | Fig 7a |

a) For the radar plot (Fig. 7i), Rc.t., DCIR, and Tortuosity were inverted so that higher values indicate better performance, and then normalized to AFPA (AFPA = 1).

**References**

[1]	Z. Fan, and S G. Advani, “Rheology of muliwall carbon nanotube suspensions,“ *Journal of Rheology* 51 (2007): 585-604, https://doi.org/10.1122/1.2736424

[2]	Y. Cao, P. Zavaterri, J. Youngblood, R. Moon, and J. Weiss, “The influence of cellulose nanocrystal additions on the performance of cement paste,“ *Cement and Concrete Composites* 56 (2015): 73-83, https://doi.org/10.1016/.cemconcomp.2014.11.008

[3]	S. Hesaraki, G. Saba, M. Shahrezaee, et al., “Reinforcing *β*‑tricalcium phosphate scafolds for potential applications in bone tissue engineering: impact of functionalized multi‑walled carbon nanotubes,“ *Scientific Reports* 14 (2024): 19055, https://doi.org/10.1038/s41598-024-68419-2

[4]	L. Sangroniz, M. Landa, M. Fernandez, and A. Santamaria, “Matching Rheology, Conductivity and Joule Effect in PU/CNT Nanocomposites,“ *Polymers* 13 (2021): 950, https://doi.org/10.3390/polym1306095

[5]	J. H. Lehman, M. Terrones, E. Mansfield, K. E. Hurst, V. Meunier, “Evaluating the characteristics of multiwall carbon nanotubes,“ *Carbon* 49 (2011): 2581-2602, https://doi.org/10.1016/j.carbon.2011.03.028

[6]	D.-W. Zhang, L. Chia, and Y. Huang, “Effect of Carboxymethyl Cellulose (CMC) Functionalization on Dispersion, Mechanical, and Corrosion Properties of CNT/Epoxy Nanocomposites,“ *Chinese Journal of Polymer Science* 41 (2023): 1277-1286, https://doi.org/10.1007/s10118-023-2928-0

[7]	J. F. Cardenas, A. Gromov, "The effect of bundling on the G’ Raman band of single-walled carbon nanotubes” *Nanotec.* 20 (2009): 465703, https://doi.org/10.1088/0957-4484/20/46/465703

[8]	X. Fan, G. Hu, B. Zhang, et al., "Crack-free single-crystalline Ni-rich layered NCM cathode enable superior cycling performance of lithium-ion batteries” Nano energy 70 (2020): 104450, https://doi.org/10.1016/j.nanoen.2020.104450

[9]	T. Liu, R. Parekh, P. Mocny, et al., "Tailored PVDF Graft Copolymers via ATRP as High-Performance NCM811 Cathode Binders” ACS Materials Lett. 5 (2023): 2594-2603, https://doi.org/10.1021/2594-2603

[10]	B. Li, W. Gong, X. Jing, and B. Zheng, “Effect of NaCl concentration on the dispersion, stability and rheologicalproperties of MWNTs by CMC,“ *Journal of Dispersion Science and Technology* 42 (2020): 2043-2052, https://doi.org/10.1080/01932691.2020.1801458

[11]	B. Zhao, D. Yin, Y. Gao, and J. Ren, "Concentration dependence of yield stress, thixotropy, and viscoelasticity rheological behavior of lithium-ion battery slurry“ *Ceramics International* 48 (2022): 19073-19080, https://doi.org/10.1016/j.ceramint.2022.03.197

[12]	J. Landesfeind, J. Hattendorff, A. Ehrl, W. A. Wall, and H. A. Gasteiger, “Tortuosity Determination of Battery Electrodes and Separators by Impedance Spectroscopy,“ *Journal of Electrochemical Society* 163 (2016): A1373-A1387, https://doi.org/10.1149/2.1141607jes

[13]	H. Oh, G.-S. Kim, J. Bang, S. Kim, and K.-M. Jeong, “Dry-processed thick electrode design with a porous conductive agent enabling 20 mAh cm^–2^ for high-energy-density lithium-ion batteries,“ *Energy & Environmental Science* 18 (2025): 645-658, https://doi.org/10.1039/d4ee04106b

[14]	A. Chauhan, E. Asylbekov, S. Kespe, and H. Nirschl, "Influence of Carbon Binder Domain on the Performance of Lithium-Ion Batteries: Impact of Size and Fractal Dimension," *Electrochemical Science Advances* 3 (2022): e2100151, https://doi.org/10.1002/elsa.202100151

[15]	T. Rajendra, A. N. Mistry, P. Patel, et al., "Quantifying Transport, Geometrical, and Morphological Parameters in Li-Ion Cathode Phases Using X‑ray Microtomography," *ACS Applied Materials & Interfaces* 11 (2019): 19933-19942, https://doi.org/10.1021/acsami.8b22758

[16]	J. Song and M. Z. Bazant, "Electrochemical Impedance of a Battery Electrode with Anisotropic Active Particles," *Electrochimica Acta* 131 (2014): 214-227, https://doi.org/10.1016/j.electacta.2014.03.013

[17]	C. Zhu, G. Fang, J. Zhou, et al., "Binder-Free Stainless Steel@Mn3O4 Nanoflower Composite: a High-Activity Aqueous Zinc-Ion Battery Cathode with High-Capacity and Long-Cycle-Life," *Journal of Materials Chemistry A* 6 (2018): 9677-9683, https://doi.org/10.1039/c8ta01198b

[18]	B. Zhao, D. Yin, Y. Gao, and J. Ren, “Temperature‑dependent rheological behavior of cathode slurry for lithium‑ion battery under steady and dynamic tests,“ *Korea-Australia Rheology Journal* 35 (2023): 191-201, https://doi.org/10.1007/s13367-023-00064-z

[19]	I. Srivastava, D. S. Bolintineanu, J. B. Lechman, S. A. Roberts, “Controlling Binder Adhesion to Impact Electrode Mesostructures and Transport,“ *ACS Appl Mater Interfaces* 12 (2020): 34919-34930, https://doi.org/10.1021/acsami.0c08251

[20]	Y. Wang, A. S. Mijailovic, et al., “ Promoting electrochemical rates by concurrent ionic-electronic conductivity enhancement in high mass loading cathode electrode,“ *Energy Storage Mater.* 71 (2024): 103546, https://doi.org/10.1016/j.ensm.2024.103546

[21]	P. Minnmann, L. Quillman, S. Burkhardt, F. H. Richter, J. Janek, “ Quantifying the Impact of Charge Transport Bottlenecks in Composite Cathodes of All-Solid-State Batteries,“ *J. Electroche. Soc.* 168 (2021): 040537, https://doi.org/10.1149/1945-7111/abf8d7

[22]	J. Ali, T. J. Embleton, J. H. Choi, et al., “Overcoming Through-Plane Resistance in Lithium-Ion Battery Cathode Electrodes via the Application of Trace High-Aspect-Ratio Carbon Nanofiber Carbon Additives with Carbon Nanotube-Coated LiNi_0.8_Co_0.1_Mn_0.1_ O_2_,” *ACS Appl. Energy Mater.* 7 (2024): 10134-10148, https://doi.org/10.1021/acsaem.4c02269
